# Bio‐energetic modeling of medium‐sized cetaceans shows high sensitivity to disturbance in seasons of low resource supply

**DOI:** 10.1002/eap.1903

**Published:** 2019-05-16

**Authors:** Vincent Hin, John Harwood, André M. de Roos

**Affiliations:** ^1^ Institute for Biodiversity and Ecosystem Dynamics University of Amsterdam 1090 GE Amsterdam The Netherlands; ^2^ Centre for Research into Ecological and Environmental Modelling University of St Andrews St Andrews Fife KY16 9LZ United Kingdom; ^3^ Institute for Biodiversity and Ecosystem Dynamics University of Amsterdam 1090 GE Amsterdam The Netherlands

**Keywords:** cetacean life history, Dynamic Energy Budget model, *Globicephala melas*, lifetime reproductive output, marine mammals, population consequences of disturbance, vital rates

## Abstract

Understanding the full scope of human impact on wildlife populations requires a framework to assess the population‐level repercussions of nonlethal disturbance. The Population Consequences of Disturbance (PCoD) framework provides such an approach, by linking the effects of disturbance on the behavior and physiology of individuals to their population‐level consequences. Bio‐energetic models have been used as implementations of PCoD, as these integrate the behavioral and physiological state of an individual with the state of the environment, to mediate between disturbance and biological significant changes in vital rates (survival, growth, and reproduction). To assess which levels of disturbance lead to adverse effects on population growth rate requires a bio‐energetic model that covers the complete life cycle of the organism under study. In a density‐independent setting, the expected lifetime reproductive output of a single female can then be used to predict the level of disturbance that leads to population decline. Here, we present such a model for a medium‐sized cetacean, the long‐finned pilot whale (*Globicephala melas*). Disturbance is modeled as a yearly recurrent period of no resource feeding for the pilot whale female and her calf. Short periods of disturbance lead to the pre‐weaned death of the first one or more calves of the young female. Higher disturbance levels also affect survival of calves produced later in the life of the female, in addition to degrading female survival. The level of disturbance that leads to a negative population growth rate strongly depends on the available resources in the environment. This has important repercussion for the timing of disturbance if resource availability fluctuates seasonally. The model predicts that pilot whales can tolerate on average three times longer periods of disturbance in seasons of high resource availability, compared to disturbance happening when resources are low. Although our model is specifically parameterized for pilot whales, it provides useful insights into the general consequences of nonlethal disturbance. If appropriate data on life history and energetics are available, it can be used to provide management advice for specific species or populations.

## Introduction

The increase of human activity in the marine environment has led to concern about the effects of disturbance on marine mammals (Halpern et al. 2008, DeRuiter et al. 2013, Maxwell et al. 2013, Fleishman et al. 2016, Parsons 2017). Several sources of disturbance (ship traffic, seismic surveys, military sonar) can lead to a variety of responses and impacts on marine mammals, such as decrease/cessation of feeding, avoidance behavior, temporary and permanent effects on hearing, and death (National Research Council 2003). The use of military sonar and the stranding of whales and dolphins has received considerable attention (Cox et al. 2006, Parsons et al. 2008, Tyack et al. 2011, Parsons 2017). While the link between disturbance and its (short term) effect on behavior, feeding, and health of individuals is becoming more apparent (Miller 2012, Sivle et al. 2012, DeRuiter et al. 2013, Christiansen and Lusseau 2015, Friedlaender et al. 2016), assessing the (long‐term) population consequences is challenging and involves many uncertainties. These arise from (among others) the inaccessibility of the marine environment and the species in question, uncertainty about many life‐history parameters and processes, the difference in timescale between a disturbance event and its consequences for populations, and the lack of information about behavioral responses that might aggravate or compensate the effect of a disturbance (Harwood and Stokes 2003, National Research Council 2005).

The PCoD (population consequences of disturbance) framework is a conceptual model to connect disturbance to its population‐level consequences by means of a number of transfer functions (New et al. 2014, Harwood et al. 2016, Pirotta et al. 2018a). These transfer functions sequentially tie the properties of disturbance to behavioral changes, life history functions, vital rates, and population effects. Although the precise nature of many of these transfer functions is unknown, it is likely that they are highly context‐dependent (National Research Council 2005, Friedlaender et al. 2016). For example, disturbance will have a different effect on lactating females in a resource‐poor environment than on non‐lactating females in a resource‐rich environment. This context dependency calls for an approach that includes the state of an individual (e.g., energy reserve, reproductive status) and the state of the environment (e.g., resource density, presence of predators) to mediate between disturbance and biologically significant changes in vital rates. Bio‐energetic models represent such an approach and have been used to assess the population consequences of disturbance for a variety of marine mammal species (New et al. 2013, Villegas‐Amtmann et al. 2015, Costa et al. 2016, McHuron et al. 2016, Pirotta et al. 2018b). However, the amount and quality of data needed to parameterize and validate such models are unavailable for many species (Harwood et al. 2016).

Bio‐energetic models that assess the effect of disturbance on marine mammals focus on female life history and, in most cases, only take into account a single reproductive cycle (Braithwaite et al. 2015, Christiansen and Lusseau 2015, Villegas‐Amtmann et al. 2015, McHuron et al. 2016, Pirotta et al. 2018b). From an energetics perspective, the reproductive period, and especially lactation, is the most demanding part of female life history and also of considerable importance for population growth. However, in order to assess under which conditions disturbance leads to negative population growth rates, it is necessary to model female life history and energetics across the entire lifespan (Villegas‐Amtmann et al. 2017, McHuron et al. 2018, Pirotta et al. 2018a). In the absence of any density dependence, population decline will occur if the expected lifetime reproductive output of a single female (*R*_0_) is smaller than 1 (counting females only; Caswell 2001). Provided that male density does not influence pregnancy rates of females, accounting for males only becomes important in the presence of density dependence. Therefore, it is possible to gain insight about the population consequences of disturbance in the absence of density‐dependence by simply evaluating the expected lifetime reproductive output of a single female.

Here we present a generic bio‐energetic model for a marine mammal life history to assess the population consequences of disturbance in relation to environmental resource availability. The model describes the entire life‐span of a female individual. In addition, we follow calf survival and development until weaning. Although the structure of the model is general enough to describe the life history of any (marine) mammal species, we parameterize and tailor this model for long‐finned pilot whales (*Globicephala melas*), a medium‐sized cetacean in which long‐distance migration is absent and feeding occurs continuously throughout the year. Pilot whales are highly social odontocetes that perform deep dives to hunt for cephalopods and several fish species (Desportes and Mouritsen 1993, Aoki et al. 2017, Isojunno et al. 2017). The choice for pilot whales is motivated by the relatively large amount of data on pilot whale bio‐energetics and life history processes (The International Whaling Commission 1993) and the availability of observations on the response of this species to (sound) disturbance (Wang and Yang 2006, Dolman et al. 2010, Miller 2012, Sivle et al. 2012, Wensveen et al. 2015, Isojunno et al. 2017). When sufficient data are available, the model could easily be parameterized for other species, including species that make long‐distance migrations with interrupted feeding (Villegas‐Amtmann et al. 2015). With this model, we aim to understand how disturbance affects reproductive abilities of the female and survival of the female and her calves, as integrated in the expected lifetime reproductive output. We assess the consequences of a yearly recurrent disturbance period that can vary in duration and timing of onset within the year. The model assumes that individual pilot whales (both the female and her calves) can behaviorally compensate for disturbance by an increase in feeding effort when body condition decreases and sufficient resources are available. We show how a number of life‐history characteristics change with increasing disturbance intensity. Furthermore, we outline how the effect of disturbance depends on environmental resource availability and its seasonal variation.

## Model Formulation

We use the Dynamic Energy Budget (DEB) model from De Roos et al. (2009) to specify how a female pilot whale individual allocates energy assimilated from resource feeding to the four energy consuming processes of (1) field metabolism, (2) energetic costs of growth in body size, (3) costs for fetal development (when pregnant), and (4) lactation costs (when lactating). In addition to resource feeding, each calf derives energy from milk feeding, while it only spends energy on field metabolism and body size growth. Both the female and each of her calves can store energy in a “reserve compartment,” which functions as a buffer for incoming and outgoing energy flows (De Roos et al. 2009, Kooijman 2010). This reserve compartment primarily represents fat tissue, which in pilot whales is mainly stored internally (in and around visceral organs and muscle tissue), but also in blubber (Lockyer 1993, 2007). The energy stored in the reserve compartment is quantified by “reserve mass” (*F*, in kg). The amount of reserve mass is an important indicator of individual health and controls several life history processes (Lockyer 2007, Miller et al. 2011). As such, reserve mass links the effects of disturbance and resource availability to survival and reproduction and, ultimately, population dynamics. To control for differences in absolute size during the female's lifetime, we use relative reserve mass (reserve mass over total body mass) as a measure of body condition. A good body condition is required to successfully raise a calf (pregnancy and lactation) and a poor body condition compromises survival and decreases life expectancy. The joint outcome of survival and reproduction of the female is summarized by the expected lifetime reproductive output (*R*_0_). Specifically, *R*_0_ is defined as the expected number of weaned calves that a single female will produce from her weaning age onward. Weaning, instead of birth, was chosen as a starting point for the calculation of *R*_0_, because mammalian individuals only become independent from their mother at weaning. We therefore simulate the life history of the female from weaning age onward and, during lactation, we simultaneously track the life history of the calf until weaning. A detailed description of the model is outlined below and all model equations and the most important parameters, in particular the directly observable life history parameters, are listed in Table 1. A complete list of parameters and their derivation is presented in Appendix S1.

**Table 1:** Overview of most important model components

Variable, parameter	Unit	Description	Value/Function
*t*	d	Time	~
Resource			
*R*	MJ/m^3^	resource density	$\hat{R}\left(1+A\sin\left(\frac{2\pi t}{365}\right)\right)$
$\hat{R}$	MJ/m^3^	annual mean resource density	1.6–3.0
*A*	–	relative amplitude of seasonal resource variation	0, 0.15, 0.3, 0.45
Age			
*a*	d	individual age	~
τ_p_	d	time since conception	~
*T*_P_	d	gestation period	365
*T*_L_	d	lactation period	1223
*T*_D_	d	waiting period	445
Reserves			
*F*	kg	reserve mass	~
	kg	pregnancy threshold	ρ_*s*_ *W* + *F*_neonate_
*F*_neonate_	kg	amount of reserves to create a newborn individual	61.45
	–	body condition	*F*/*W*
ρ	–	target body condition	0.3
ρ_*s*_	–	starvation threshold	0.15
Structural length			
*l*(*a*)	cm	length–age relationship, free‐living individual	$l_{\infty }-\left(l_{\infty }-l_{b}\right)e^{-ka}$
*l*_*b*_	cm	length at birth	177
*l*_∞_	cm	asymptotic length	450
*k*	d^−1^	Von Bertalanffy growth rate	0.00045
*l*_*p*_ (τ_*p*_)	cm	length–age relationship fetus	$l_{b}\frac{\tau_{p}}{T_{P}}$ for 0 ≤ τ_*p*_ ≤ *T*_*P*_
Structural mass			
*S* (*l*)	kg	structural mass–length relationship	$\omega_{1}l{\left(a\right)}^{\omega_{2}}$
ω_1_	kg/cm^ω2^	mass–length scaling constant	8.5 × 10^–5^
ω_2_	–	mass–length scaling exponent	2.6
Total body mass			
*W* (*S*,* F*, τ_*p*_)	kg	total body mass	$\begin{array}{c} \left\{\begin{array}{cc} S+F+S\left(l_{p}\left(\tau_{p}\right)\right) & \text{pregnant}\\ S+F & \text{otherwise} \end{array}\right. \end{array}$
*W*_M_(*S*,* F*, τ_*p*_)	kg	maintenance body mass	$\left\{\begin{array}{cc} S+\theta_{F}F+S\left(l_{p}\left(\tau_{p}\right)\right) & \text{pregnant}\;\\ S+\theta_{F}F & \text{otherwise} \end{array}\right.$
θ_*F*_	–	relative maintenance costs of reserves	0.2
Energetic rates			
*I*_*R*_(*a*, *R*, *S*, *F*, *W*)	MJ/d	energy assimilation from resource feeding	$\phi_{R}RS^{2/3}\frac{1}{1+e^{-\eta \left(\rho W/F-1\right)}}\frac{a^{\gamma }}{T_{R}^{\gamma }+a^{\gamma }}$
$I_{L}\left(a,S,F,W,F_{m},W_{m}\right)$	MJ/d	energy assimilation from milk feeding	$\begin{array}{cc} & \phi_{L}S^{\frac{2}{3}}\frac{1}{1+e^{-\eta \left(\frac{\rho W}{F}-1\right)}}\min\left(1,{\left[\frac{1-\frac{a-T_{N}}{T_{L}-T_{N}}}{1-\xi_{c}\frac{a-T_{N}}{T_{L}-T_{N}}}\right]}_{+}\right)\times \\ & {\left[\frac{\left(1-\xi_{m}\right)\left(F_{m}-\rho_{s}W_{m}\right)}{\left(\rho -\rho_{s}\right)W_{m}-\xi_{m}\left(F_{m}-\rho_{s}W_{m}\right)}\right]}_{+} \end{array}$
*C*_*M*_(*W*_*M*_)	MJ/d	field metabolic costs	$\sigma_{M}W_{M}^{3/4}$
*C*_*G*_(*l*)	MJ/d	structural growth costs	$\sigma_{G}\omega_{1}k\left(l_{\infty }-l\right)\omega_{2}l^{\omega_{2}-1}$
*C*_*P*_(*τ*_*p*_)	MJ/d	fetal development costs	$\sigma_{G}\omega_{1}\omega_{2}{\left(\frac{l_{b}}{T_{P}}\right)}^{\omega_{2}}{\tau_{p}}^{\omega_{2}-1}$ for $0\leq \tau_{p}\leq T_{P}$
*C*_*L*_(*F*, *W*, *a*_c_, *S*_c_, *F*_c_, *W*_c_)	MJ/d	lactation costs	$I_{L}\left(a_{c},S_{c},F_{c},W_{c},F,W\right)/\sigma_{L}$
Mortality			
*D*(*a*)	d^−1^	age‐dependent mortality rate	$\alpha_{1}e^{-\beta_{1}a}+\alpha_{2}e^{\beta_{2}a}$
*D*_*s*_ (*F*,* W*)	d^−1^	starvation‐induced mortality rate	$\mu_{s}\left(\frac{\rho_{s}W}{F}-1\right)$if *F* < ρ_*s*_ *W*

*Notes*

Variables are indicated with ~. A + subscript indicates that only positive values are used, i.e., $f_{+}=\max\left(f,0\right)$. Indices c and m indicate that a variable belongs to the calf and the female, respectively.

### Individual state

All energetic rates (Table 1) depend on resource density (*R* in MJ/m^3^) and/or on the state of the individual. Individuals are characterized by age (*a*, d), structural size (length *l*, in cm, and structural mass *S*, in kg), reserve mass (*F*, in kg), total and maintenance body mass (*W* and *W*_M_ in kg) and reproductive status. The structural component of an individual includes all tissue that cannot be mobilized to fuel energetic needs (i.e., growth, metabolism, gestation and lactation), such as bones and vital organs (Kooijman 2010). In contrast, assimilated energy or energy mobilized from the reserves is used for such purposes. Structural mass changes due to structural growth. Dynamics of reserve mass are given by the difference between total energy assimilation and total energy expenditure (see reserve mass dynamics). The reproductive status of the female can be either “resting,” “waiting,” “pregnant,” “lactating,” or “waiting and lactating.”

### Structural growth

Growth in structural size of mammals is best represented by a demand type of growth, in which structural growth rate and asymptotic structural size do not vary with the amount of assimilated energy (Sebens 1987). Instead, structural growth poses a certain energy demand on the environment. Based on pilot whale data in Bloch et al. (1993), we use a Von Bertalanffy relationship between structural length and age for free‐living individuals with parameters length at birth *l*_b_ = 177, asymptotic length *l*_∞_ = 450, and Von Bertalanffy growth rate *k* = 0.00045 (Table 1; Appendix S1: Fig. S1). The length–age relationship of a fetus (*l*_*p*_(τ_*p*_)) is approximated by a linear function of time since conception, *τ*_*p*_, such that fetuses reach length at birth (*l*_b_) when the gestation period (*T*_*P*_ = 365 d) is due (Table 1, Bloch et al. 1993). Structural mass (*S*) is related to structural length by a power function. Total body mass, *W*, equals the sum of structural mass *S* and reserve mass *F*. For pregnant females, total body mass also includes the structural mass of the fetus $S(l_{p}\left(\tau_{p}\right))$. Body condition, or relative reserve mass, is given by *F*/*W*.

### Resource feeding

Rate of energy assimilation from resource feeding (*I*_R_(*a*, *R*, *S*, *F*, *W*)) is composed of four parts. The first part describes a linear functional response of resource density (ϕ_*R*_ *R*). A linear functional response was assumed based on the proposition that handling and/or digestion time should rarely limit resource intake rate for species such as pilot whales that make short dives to find their food and spend most of their time at the surface (Baird et al. 2002, Heide‐Jorgensen et al. 2002, Isojunno et al. 2017). Quantities such as assimilation and conversion efficiencies, as well as resource encounter rate and catch probability are captured in resource density *R* (in MJ/m^3^) and multiplication with scalar ϕ_*R*_ only occurs to relate this resource density to the rate of resource assimilation. Because *R* and ϕ_*R*_ only enter the model through their product (ϕ_*R*_ *R*), the value of ϕ_*R*_ on its own is arbitrary and will not affect model dynamics. The resource density *R* should be interpreted as the amount of assimilated energy per unit of volume that is available in the environment, while the product ϕ_*R*_ *R* is the assimilated energy acquired per day per unit of *S* ^2/3^. The second part of *I*_*R*_ describes the scaling of resource ingestion with structural whale mass to the two‐thirds power, *S* ^2/3^ (Kooijman 2010). Taken together, the product ϕ_*R*_ *RS* ^2/3^ accounts for the maximum resource assimilation rate per individual whale, at resource density *R*.

The third part of the resource assimilation rate simulates an increase in an individual's feeding effort with decreasing body condition *F*/*W* (first fraction of *I*_*R*_ in Table 1). The feeding effort operates as a negative feedback of body condition to ensure that the reserve mass does not grow out of bounds under favorable conditions (high resource availability or low energy expenditure). Similarly, the increase in feeding effort when body condition is low can be seen as a behavioral compensation response to disturbance or low resource availability. Feeding effort is implemented as a sigmoidal decreasing function (0–1) of body condition that equals 0.5 at the target body condition ρ (see Appendix S1: Fig. S1b). Lockyer (1993) notes that body condition of pilot whales is independent of age and reproductive status and finds no evidence for an energy storage strategy to fulfil energetic demands of reproduction. Consequently, we use a constant target body condition of 0.30 (Appendix S1). The last component of *I*_*R*_ modifies the age‐dependency of resource assimilation to simulate the observation that young individuals are inexperienced resource foragers and that foraging skills increase with age (Lockyer 2007, Isojunno et al. 2017). This component increases from 0 at birth and asymptotically approaches 1 with increasing age (Appendix S1: Fig. S1c).

### Milk consumption

Like resource feeding, milk assimilation *I*_L_(*a*, *S*, *F*, *W*, *F*_m_, *W*_m_) of a calf scales with the two‐thirds power of its structural mass (*S* ^2/3^), and is furthermore proportional to the lactation scalar ϕ_L_. Also the feeding effort as a function of calf body condition decreases milk suckling in the same way as it modifies resource assimilation (Table 1). Contrary to resource assimilation, milk consumption decreases with calf age. Beyond the first year of lactation, milk consumption decreases such that it becomes zero at the age of weaning, *T*_L_.

The female also regulates milk provisioning to the calf in a manner that depends on her own body condition (last component of function *I*_L_ in Table 1). This component ensures that milk provisioning equals 1 if the female's body condition is equal to the target body condition ρ. When the female's body condition declines, milk provisioning is decreased and when her body condition reaches the starvation body condition threshold ρ_*s*_, the female ceases milk supply altogether (Appendix S1: Fig. S1e). At weaning age *T*_L_ the calf becomes independent of the focal female and is no longer tracked during simulations. At any time before *T*_L_, the milk supply will be interrupted only when the mother's body condition falls below ρ_s_. The possibility of early weaning resulting from good body condition of mother and calf is not modeled explicitly. However, due to the dependence of lactation on female and calf body condition, milk consumption will be limited under these circumstances. Based on data in Martin and Rothery (1993), we adopt *T*_L_ = 1,223. The parameters ϕ_L_ and ρ_s_ were derived to be 2.7 and 0.15, respectively (see Appendix S1).

### Energetic costs

Energetic costs (in MJ/d) are denoted by *C*_*i*_ and are comprised of $C_{M}\left(W_{M}\right)$, field metabolic costs depending on the maintenance body mass (*W*_*M*_); $C_{G}\left(S\right)$, costs of growth in structural size; $C_{P}\left(\tau_{p}\right)$, fetal development costs depending upon time since fertilization; and $C_{L}\left(F,W,a_{c},S_{c},F_{c},W_{c}\right)$, lactation costs depending upon both age and body condition of the calf (subscript c indicates a calf variable), in addition to the body condition of the mother (Table 1). Field metabolic costs include metabolic costs of maintenance and daily routine activity. We assume that cost of transport/movement are covered by field metabolic costs and are independent of the feeding effort. We furthermore ignore seasonal variation in field metabolic rate, or any dependency of metabolism on environmental conditions or behavioral state. Consequently, metabolic costs spend on thermoregulation are not explicitly considered, but we note that such costs could readily be incorporated in our model, provided that they can be measured with sufficient level of accuracy. Since maintenance of 1 kg of reserve mass is lower than that of 1 kg of structural mass, we introduce the maintenance body mass *W*_M_. This measure uses the proportionality constant θ_*F*_ to discount the contribution of reserve mass to maintenance costs (see Table 1) and is set to 0.2. For pregnant females, the structural body mass of the fetus is also incorporated in the maintenance body mass *W*_M_. Field metabolic costs are a σ_M_− multiple of the maintenance mass, raised to the 3/4 power following Kleiber (1975). Structural growth costs are equal to the derivative of the Von Bertalanffy growth function in structural mass multiplied with proportionality constant σ_*G*_ = 30 MJ/kg, which represents the energetic cost of producing 1 kg of structural mass, i.e., $C_{G}\left(S\right)=\sigma_{G}\frac{dS(l(a))}{\mathrm{da}}$. The same holds for fetal development costs, which are equal to the derivative of fetal structural growth multiplied by σ_*G*_, i.e., $C_{P}\left(\tau_{p}\right)=\sigma_{G}\frac{dS(l_{p}\left(\tau_{p}\right))}{d\tau_{p}}$. Note that the function *C*_*p*_ only covers the structural growth costs of the fetus, while the maintenance costs of the growing fetus are modeled by incorporating its mass into the maintenance body mass of the pregnant female. Lactation costs equal the milk provisioning rate *I*_L_, corrected by the conversion factor σ_L_ = 0.86 (Appendix S1), which accounts for both efficiency of milk production by the mother (0.90) and efficiency of milk assimilation by the calf (0.95).

### Reserve mass dynamics

Dynamics of reserve mass follow from adding and subtracting anabolic and catabolic processes and accounting for the conversion efficiency of catabolism and anabolism. Independent of individual status, reserve mass increases due to resource assimilation and decreases with field metabolic costs and somatic growth costs. For calves, milk feeding, in addition to resource feeding, increases reserve mass. For pregnant and lactating females, reserve mass decreases through fetal development and lactation costs, respectively. The conversion efficiency (ɛ_*i*_) equals ɛ_+_ if reserve dynamics are anabolic (*dF*/*da* > 0) and ɛ_−_ if reserve dynamics are catabolic (*dF*/*da* < 0). Taken together, reserve mass dynamics are described by equation (1).

(1) $$\begin{array}{c} \frac{\mathrm{dF}}{\mathrm{da}}=\left\{\begin{array}{cc} \varepsilon_{i}^{-1}\left(I_{R}\left(a,R,S,F,W\right)+I_{L}\left(a,S,F,W,F_{m},W_{m}\right)-C_{G}\left(l\right)-C_{M}\left(W_{M}\right)\right) & \mathrm{Calves}\\ \varepsilon_{i}^{-1}\left(I_{R}\left(a,R,S,F,W\right)-C_{G}\left(l\right)-C_{M}\left(W_{M}\right)-C_{P}\left(\tau_{c}\right)\right) & \mathrm{Pregnant}\;\mathrm{female}\\ \varepsilon_{i}^{-1}\left(I_{R}\left(a,R,S,F,W\right)-C_{G}\left(l\right)-C_{M}\left(W_{M}\right)-C_{L}\left(F,W,a_{c},S_{c},F_{c},W_{c}\right)\right)\; & \mathrm{Lactating}\;\mathrm{female}\\ \varepsilon_{i}^{-1}\left(\right.I_{R}\left(a,R,S,F,W\right)-C_{G}\left(l\right)-C_{M}\left(W_{M}\right) & \text{Other} \end{array}\right. \end{array}$$

### Survival and life expectancy

In order to calculate lifetime reproductive output, we track survival probabilities of the female and her calves. There are two sources of mortality that decrease survival probability: age‐dependent mortality and starvation‐induced mortality. Age‐dependent mortality applies to all individuals and consists of (1) juvenile mortality that decreases with age, (2) senescence mortality that increases with age and (3) background mortality that is constant with age (Barlow and Boveng 1991; Appendix S1: Fig. S1f; Bloch et al. 1993). These three types of age‐dependent mortality are captured by function *D*(*a*) in Table 1. This equation was fitted to data of Bloch et al. (1993) and provides an equal fit compared to an equation that describes the three forms of age‐dependent mortality as three separate terms, but requiring an additional parameter. Starvation‐induced mortality is only applied when the body condition of an individual falls below the starvation threshold (ρ_s_ = 0.15). Starvation mortality increases with declining body condition according to a hyperbolic function, with the speed of increase controlled by parameter μ_*s*_ (Appendix S1: Fig. S1g; De Roos et al. 2009).

Depending on the purpose of the simulation, life expectancy is either fixed or determined randomly. A fixed life expectancy is used when illustrating the consequences of disturbance across the entire life of the focal female. In this case, a life expectancy at birth of 60 yr is used (Bloch et al. 1993, Lockyer 2007). When only applying the age‐dependent mortality as described above, this corresponds to a survival threshold of 2.266 × 10^−7^. Therefore, the female is considered dead when her survival probability falls below this threshold. Because the female is initiated at weaning, instead of at birth, she will reach the starvation threshold at an age that slightly exceeds 60 yr, in absence of starvation mortality. However, when survival is also decreased by starvation mortality, the female's life expectancy is decreased since she will cross the survival threshold at a younger age (Appendix S1: Fig. S1h). Even if the female's body condition recovers above ρ_s_, she will have a lower age‐dependent survival curve compared to the situation if starvation had not occurred. Consequently, the remaining life expectancy of a female that experiences starvation will be decreased. The threshold survival probability of 2.266 × 10^−7^ is also used for calves, which implies that calves will only die before they reach weaning age in case of a substantial amount of starvation mortality.

A randomly determined life expectancy is used when calculating the expected lifetime reproductive output (*R*_0_) of the focal female. The calculation of *R*_*0*_ requires using the mean life expectancy of a female, in contrast to a fixed life expectancy of 60 yr. Besides this, the random event of a calf death introduces variation in the timing of the next reproduction, which essentially creates an infinite number of possible life histories for the female. An accurate estimate of *R*_0_ requires averaging female reproductive output over all those possible life histories, or at least over a substantial subset. The randomly determined life expectancy is implemented by assigning a random number between zero and one to the female at weaning age and to each calf at birth. Subsequently, the individual (either the female or her calf) dies when its survival probability falls below this threshold value (De Roos et al. 2009). Both *R*_0_ and mean life expectancy can then be calculated by averaging the reproductive output and age at death of a sufficiently large number of life history simulations. For both random and fixed life expectancies, we additionally assume that death is certain if body condition falls below 0.005. However, for most, if not all values of the survival threshold, an individual that suffers from starvation will die long before its body condition reaches this lower threshold value.

### Reproduction

Reproduction is initiated when the female has accrued sufficient reserves to cover the energetic costs of fetal growth and development, on top of the reserve mass that the female needs to offset starvation (ρ_s_ *W*). The costs for fetal growth and development are collectively referred to as *F*_neonate_, which has two components. The first component, $\frac{\sigma_{G}\omega_{1}{l_{b}}^{\omega_{2}}}{\varepsilon_{-}}$, accounts for the growth costs of the structural mass of the fetus, expressed in reserve mass of the female (hence the division by ɛ_−_). The second component, $\frac{\rho_{s}\omega_{1}{l_{b}}^{\omega_{2}}}{\left(1-\rho_{s}\right)}$, accounts for the amount of reserves that the female transfers to the calf at birth. This implies that fetuses are assumed to grow in structural mass only and at birth they receive an amount of reserves that equals the starvation reserve mass threshold for neonates (ρ_*s*_ *W*_*b*_). This transfer of reserves at birth is assumed to occur without any overhead costs. The component *F*_neonate_ is only used to determine the onset of reproduction, as it is not necessarily equal to the cost of fetal development during gestation. These costs vary depending on how much energy the female mobilizes from her reserve mass, which incurs a conversion efficiency of $\frac{\varepsilon_{-}}{\varepsilon_{+}}<1$. We refer to the sum of *F*_neonate_ and ρ_s_ *W* as the “pregnancy threshold” (Table 1).

A non‐pregnant and non‐lactating female is assigned to the ‘resting’ state if her reserve mass is below the pregnancy threshold. When the reserve mass of the female crosses the threshold (i.e., *F* > ρ_s_ *W* + *F*_neonate_) she is assigned the ‘waiting’ state, as she does not become pregnant immediately. Instead, she awaits implantation. The waiting period (*T*_*D*_) lasts 445 d, which is based on the assumption of one ovulation per year and a chance of successful insemination of 0.82 (see Appendix S1). Pregnancy starts when the waiting period is due, irrespective of the female's reserve mass at that point in time. This assumption is required to prevent females from never starting pregnancy in case resource seasonality and yearly disturbance events decrease the female's reserve mass below the pregnancy threshold during the waiting period of 445 d. Pregnancy lasts for *T*_P_ = 365 d. Lactation is initiated after the birth of the calf and lasts *T*_L_ = 1,223 d, unless the calf dies, or the mother stops milk provisioning due to poor body condition. A lactating female can reinitiate pregnancy (enter the waiting period) if her reserve mass is above the pregnancy threshold, but only when she is within the final *T*_P_ = 365 d of the lactation period. This additional condition prevents the occurrence of two calves that simultaneously feed from the mother. Taken together, the female will be in one of the following reproductive classes: resting, waiting, pregnant, lactating, and simultaneously waiting and lactating. In principle, the female could also be pregnant and lactating simultaneously, although the fact that the waiting period is longer than the gestation period (*T*_D_ > *T*_P_) prevents this from happening. The shortest possible interval between a weaning event and the next onset of pregnancy is hence 445 − 364 = 81 d. Given that the calf survives until the end of the lactation period, the shortest possible interval between two birth events (the inter‐birth interval) is 4.57 yr (81 + 365 + 1,223 d).

### Resource dynamics and disturbance

Environmental resource density *R* fluctuates around a yearly mean value $\hat{R}$ with a seasonal pattern (period is 365 d) and an amplitude of *A* (0–1) that is relative to $\hat{R}$ (Table 1). The seasonal fluctuations in resource density are used as a simple representation of the spatiotemporal variation in food availability that is characteristic of the marine environment. Such variation might arise from seasonal migration of prey, seasonal changes in ocean productivity or the relatively short, semelparous life histories of several cephalopod species that form the main prey of long‐finned pilot whales (Desportes and Mouritsen 1993, Boyle et al. 1996). With seasonal variation (*A *>* *0), resource density is at its mean value $\hat{R}$ and increasing on the first day of each year (*t* = 0, 365, etc.), which we arbitrary label as the middle of spring. Since the simulation starts at *t* = 0, the simulated life of the female is therefore initiated in the middle of spring. This choice is not based on any data and should be regarded as the most conservative option, because the female faces a period of high resource availability immediately after initiation. With seasonal variation, the resource density peaks at *t* = 91 d, which we call the middle of summer, and reaches its minimum in the middle of winter, at *t* = 273 d.

Disturbance is modeled as a yearly recurrent period of no feeding that lasts a certain number of consecutive days per year. Consequently, disturbance is characterized by a disturbance duration and a starting date. Concerning the latter, we distinguish between summer (starting when (*t* mod 365) = 91 d) and winter disturbance (starting when (*t* mod 365) = 273). The duration of disturbance is varied upon analysis and refers to the number of consecutive days per year for which resource ingestion rates of the female (and, if present, the calf) are set to zero for 24 h/d. Real‐world disturbance scenarios are likely to be less extreme, and we emphasize that long disturbance durations represent a worst‐case scenario (see *Discussion*). Lactation is still possible during disturbance.

### Model analysis

Model equations are implemented in the Escalator Boxcar Train (EBT; De Roos 1988) software package (*available online*).^1^ This package solves a set of ordinary differential equations (ODEs) describing the survival, growth, and reproduction of the female and her calves. The integration of these ODEs is interrupted by events related to the onset of reproduction (when the pregnancy threshold is crossed), initiating of pregnancy, birth, weaning, and death. Model output was processed and plotted using R software (version 3.5.1) with the ggplot2 package (version 3.1.0; Wickham 2016, R Core Team 2017). The EBT model implementation file and R code for plotting are available online (see *Data Availability*).

We start off by illustrating the consequences of an increase in disturbance period on the life history of the female pilot whale and her calves by using the fixed life expectancy of 60 yr. In the same setting, we explore the consequences of seasonal variation in resource density and differences in the timing of disturbance (summer vs. winter). With the fixed life expectancy, the number of weaned calves during the female's lifetime provides an upper estimate of the reproductive capacity. To arrive at a representative estimate of *R*_0_, we use the randomly determined life expectancy and average the lifetime reproductive output of the female across 1,000 simulated life histories. In addition to *R*_0_, we calculate the following life history statistics: mean age at death as a measure of life expectancy, mean percentage of calves that survive until weaning age, mean female age at first reproduction, mean female age at which first calf is weaned (age at first weaning), and the mean time between different birth events (inter‐birth interval). Basic nonparametric bootstrapping with 1,000 resamples was used to calculate the mean and 95% confidence intervals of these life history statistics. This procedure is repeated for nine different values for mean annual resource density (*R* = 1.6, 1.7, 1.8, 2.0, 2.2, 2.4, 2.6, 2.8, 3.0), four levels of resource seasonality (*A *=* *0, 0.15, 0.3, 0.45), 11 disturbance periods (0, 5, 10, 15, 20, 25, 30, 35, 40, 45, 50), and two different disturbance seasons (summer/high vs. winter/low).

## Results

### Undisturbed pilot whale life history

**Figure 1:**
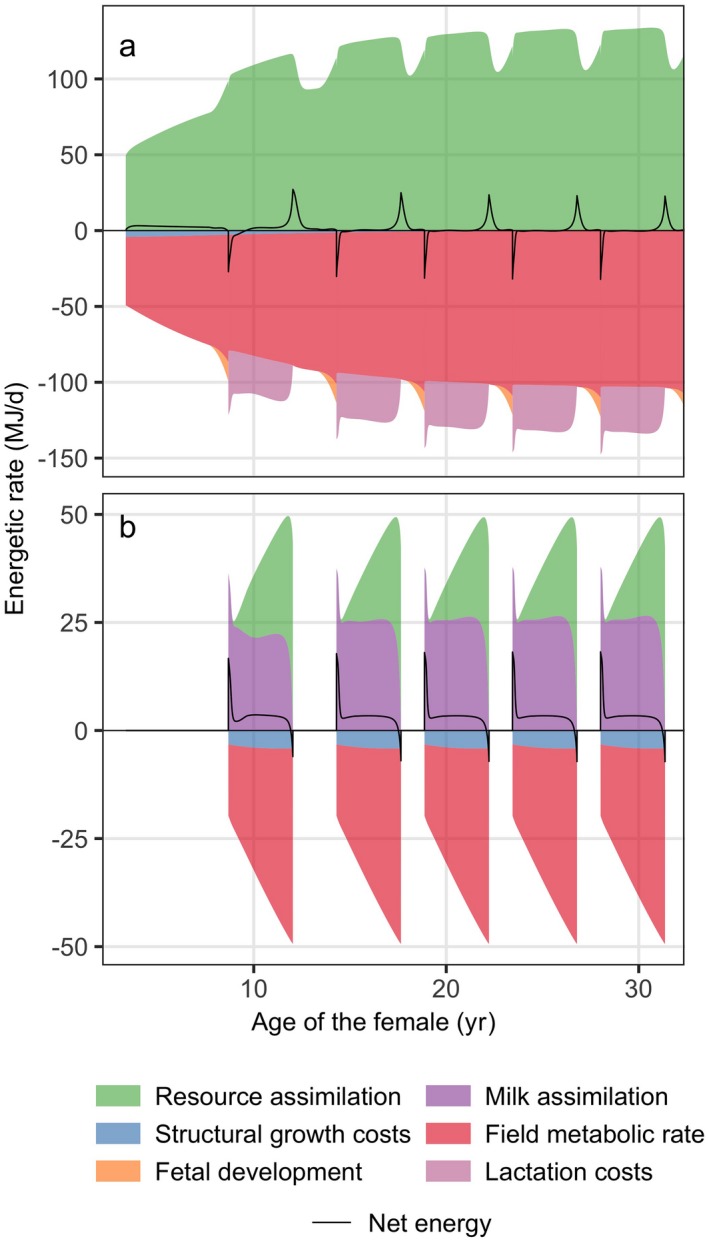
An area graph of the energetic rates of the Dynamic Energy Budget (DEB) model for (a) the female and (b) each of her calves. Rates of energy intake (resource and milk assimilation) are plotted as positive values, while rates of energy expenditure (structural growth costs, field metabolic rate, fetal development, and lactation costs) are plotted as negative values. Energetic rates are displayed in a cumulative fashion, by plotting each rate on top of the other one. Net energy represents the difference between total incoming and outgoing rates of energy. Only the first 32 yr of the female's life are plotted. Mean annual resource density $\hat{R}=1.8$ and all other parameters at default values (Appendix S1: Table S1).

Fig. 1 shows the rates of energy intake (positive values) and expenditure (negative values) of the female and her calves as a function of female age for mean constant resource density of *R *=* *1.8. The net energy intake rate represents the difference between all incoming and all outgoing energy flows and indicates whether reserve mass is increasing (net energy is positive) or decreasing (net energy is negative). Before and during the first pregnancy, the net energy rate of the female is slightly positive due to growth in structural mass and accumulation of reserve mass (Fig. 1a). When the female is fully grown (age > 15 yr), the net energy rate equilibrates at zero, except at the onset and end of lactation. In the initial phase of lactation, net energy is negative (reserve mass decreases) and when the female recovers from lactation, net energy becomes positive (reserve mass increases again). Overall, field metabolic rate is the largest source of energy expenditure and structural growth costs are relatively minor. Costs of lactation far outweigh costs of fetal development (Fig. 1a). Energetics of the calf (Fig. 1b) show an initial dependence of milk and increasing contribution of resource assimilation during lactation.

**Figure 2:**
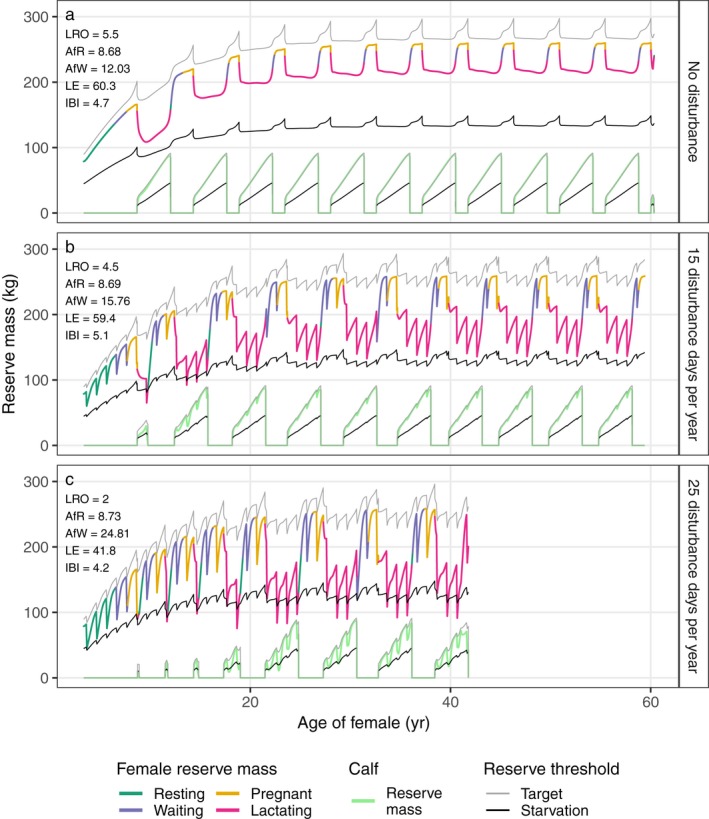
Reserve mass of the female and her calves as a function of female age for different disturbance periods (0, 15, and 25 d/yr). Female reserve mass is colored according to reproductive status (as indicated). A non‐pregnant and non‐lactating female is coined “waiting” when her reserve mass is above the pregnancy threshold (not shown) and she awaits implantation, otherwise she is coined “resting.” Target and starvation reserve thresholds are plotted for both the female (upper lines) and calves (lower lines) and are equal to total body mass multiplied by ρ = 0.3 and ρ_s_ = 0.15, respectively. LRO, lifetime reproductive output (female offspring only); AfR, female age at first reproduction (yr); AfW, female age at which first calf is weaned (yr); LE, life expectancy (yr); and IBI, inter‐birth interval (yr). A fixed life expectancy at birth of 60 yr was used, which can only decrease due to additional starvation mortality. Mean annual resource density $\hat{R}=1.8$ and all other parameters at default values (Appendix S1: Table S1).

Female reserve mass increases with age, although high lactation costs temporarily decrease reserve mass (Fig. 2, for mean annual resource density $\hat{R}$ = 1.8). Outside lactation periods, female reserve mass approaches an equilibrium value that is slightly below the target reserve threshold, which is a constant fraction of total mass (ρ*W*). Structural mass is the main component of total mass and increases with age according to the Von Bertalanffy function (Table 1). This drives the increasing asymptotic trend of reserve mass with age. Furthermore, reserve mass itself also contributes to total mass and the target and starvation reserve thresholds therefore also depend on reserve mass. Effectively, an increase in reserve mass triggers an increase in the target reserve threshold, although this increase is disproportionally smaller due to the large contribution of structural mass to total mass. When pregnant, the target and starvation reserve thresholds of the female are increased due to the contribution of fetal mass to total mass. The increase in target reserve threshold will lead to a higher feeding effort and in this way cover gestation costs. Fig. 2a shows that reserve mass stays approximately constant during pregnancy, although the target reserve mass peaks due to the contribution of fetal mass.

Without disturbance, the reserve threshold for initiation of pregnancy is crossed for the first time at an age of 6.5 yr and first birth occurs at age 8.7 yr (Fig. 2a). During the first lactation period the depletion of the female's reserve mass is most pronounced, as the female's structural mass is still developing and the absolute amount of reserves she can carry is limited. Female age also affects the inter‐birth interval. After the first lactation period, the female is “resting” and initiation of pregnancy only occurs *T*_D_ = 445 d after the day she enters the waiting period (crosses the pregnancy threshold). During subsequent lactation periods the female's reserve mass stays above the pregnancy threshold and she already enters the waiting period within the last year of lactation. Consequently, there are only *T*_D_ − 364 = 81 d between the weaning of a calf and initiation of the next pregnancy. Assuming that the female survives until age 60, she is able to wean 11 calves (5.5 females on average; Fig. 2a). This maximum reproductive potential is controlled more by the duration of the different reproductive phases (waiting, gestation and lactation) than by resource density. Increasing resource density from 1.8 to 5.0 would only lead to one extra calf being successfully weaned, setting the maximum reproductive potential to 12 calves (6 females on average).

In contrast to the female, the reserve mass of each calf closely approximates the target reserve threshold (Fig. 2a). Consequently, calves have a higher body condition than the female (maximum values 0.305 vs. 0.277, respectively). Since the target reserve threshold (ρ = 0.3) is independent of individual age or reproductive status, the higher body condition of calves is an emergent property of the model and stems from the fact that calves benefit from two food sources simultaneously (resource and milk feeding). The consistently higher body condition of calves only occurs in an undisturbed environment with a constant, high resource density.

Some aspects of the bio‐energetics of an undisturbed, fully grown female and her calf are listed in Table 2. When fully grown, the female has a structural mass of 672 kg and her reserve mass varies between 217 and 257 kg., depending on whether she is pregnant, lactating, or recovering from lactation. In the latter case, the female awaits new implantation, but her reserve mass is still increasing. In comparison, if the female did not engage in reproductive activity, her reserve mass would equilibrate over time at 260 kg, but this state is never reached. Field metabolic costs are 103 MJ/d. On average, lactation increases resource assimilation rate more than pregnancy (29% vs. 8.7%) and one year of lactation is around four times more expensive than one year of pregnancy (11,476 MJ vs. 2,880 MJ), accounting for a 3 MJ/d increase in metabolic rate during pregnancy. During the first year, 91% of the calf's energy is derived from milk, and for the remainder of the lactation period, milk provides 58% of its energy requirements (66% on average for the whole period).

**Table 2:** Some aspects of the energetics of the modeled pilot whale female, living in an undisturbed, constant environment with resource density *R* = 1.8 and other parameters as default (Appendix S1: Table S1)

Quantity	Mean^a^	Increase (%)	Total^a^
Reserve mass			
At equilibrium	260	0 (reference value)	
Recovering^b^	245 (233–254)	−5.7% (−10 to −2.3)	
During pregnancy	257 (253–258)	−1.2% (−2.7 to −0.8)	
During lactation	217 (213–248)	−16% (−18 to −4.6)	
Resource assimilation			
For metabolism	103	0 (reference value)	
Recovering^b^	118 (111–126)	15% (7.8–22)	
During pregnancy	112 (106–123)	8.7% (2.9–19)	
During lactation	133 (116–134)	29% (13–30)	
Pregnancy costs			
Structural growth fetus	4.9 (0–13)		1,787
Metabolic rate during pregnancy	106 (104–111)	2.9% (1.0–7.7)	
Lactation costs			
First year	31 (29–44)		11,476
Whole period	30 (0.2–44)		36,210
Calf milk assimilation			
First year	27 (25–38)		9,870
Whole period	25 (0.2–38)		31,140
Calf resource assimilation			
First year	2.7 (0–7.1)		1,017
Whole period	13 (0–42)		16,338

*Notes*

The female is fully grown (growth costs are zero) with a structural mass of 672 kg.

Mean (minimum–maximum) values for reserve mass are in kg and other values are rates in MJ/d.

a Total rates (in MJ) are integrated over the relevant period.

b The recovering female is waiting to become pregnant again, but her reserve mass still increases from the previous lactation period.

### Effect of disturbance

The first effect of disturbance, defined as a complete cessation of resource feeding, is reduced survival or death of calves born to young females. Depending on the duration of disturbance, the first one or more calves of the female die before weaning. We illustrate this effect of disturbance for a yearly recurrent disturbance period of 15 consecutive days in Fig. 2b. Here, the disturbance event within the first lactation period in the life of the female leads to starvation of both calf and female (reserve masses drop below their starvation thresholds in Fig. 2b), resulting in death of the calf before weaning. During lactation of the second calf, the female experiences two minor starvation events during two recurrent disturbances and both the calf and the female survive. As a consequence of 15 d of lost foraging per year, the age of the female when she weans her first calf (age at first weaning) increases from 12 to 15.8 yr. The increased mortality experienced by the female during the first two lactation periods only leads to a minor decrease in female life expectancy, compared to the scenario without disturbance (59.4 vs. 60.3 yr).

Besides causing pre‐weaned death of calves of young females, 15 disturbance days per year also increases the inter‐birth interval (Fig. 2b). Except for the last four lactation periods, the female only crosses the pregnancy threshold when lactation is finished. During the last four lactation periods, she does cross the pregnancy threshold in the last year of lactation, but not immediately on the first day of this last year. The combined effect of the early death of the first calf and the prolonged time periods between birth and weaning events is that 15 d of recurrent disturbance reduces the reproductive potential of the female to nine calves (4.5 females on average).

Increasing the disturbance period leads to the pre‐weaned death of multiple calves and can substantially shorten female life expectancy. We illustrate this effect of longer disturbance durations in Fig. 2c with a yearly recurrent disturbance of 25 d (25 consecutive days of lost foraging per year). This leads to pre‐weaned death of the first four calves (age at first weaning increases to 24.8 yr) and a reduction in life expectancy to 42 yr. Starvation of the female mainly occurs during the lactation periods of the calves that do survive, while the pre‐weaned deaths of the first 4 calves only incur very short starvation periods. Age at first reproduction is only moderately affected by disturbance, and the average inter‐birth interval actually decreases, because the early death of a calf allows the female to give birth to the next calf sooner.

### Resource seasonality and the effects of disturbance

**Figure 3:**
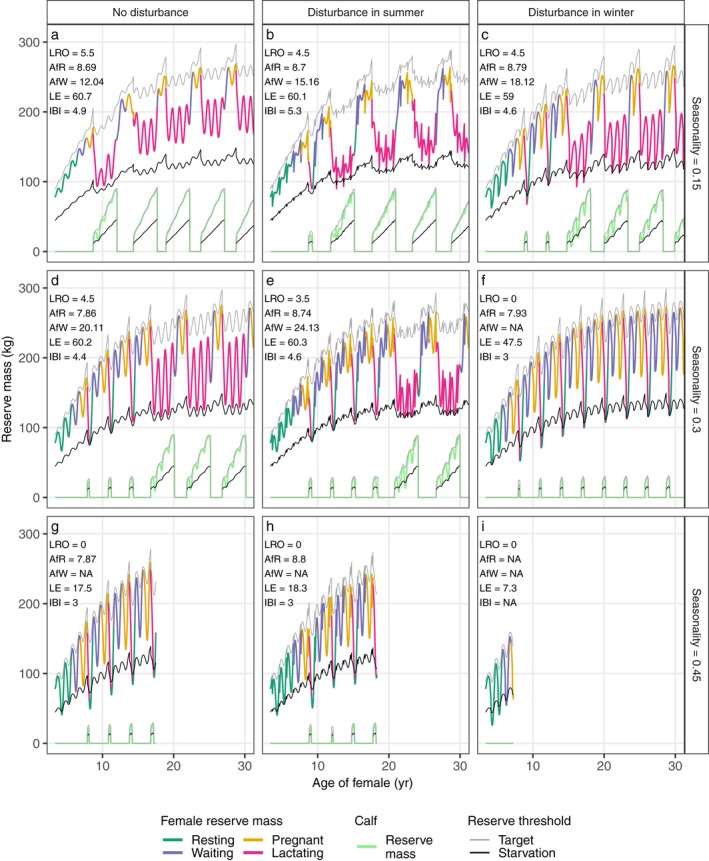
Reserve mass of the female and her calves as a function of female age for different resource amplitudes (0.15, 0.30, and 0.45, in different rows) and with either no disturbance (left columns), 15 d of summer disturbance (middle columns) or 15 d of winter disturbance (right columns). Lines, life history statistics, color‐coding, and other parameters as in Fig. 2. Only the first 30 yr of female life are plotted as the female is fully grown by that age and patterns of reserve dynamics do no longer change. As in Fig. 2, a fixed life expectancy of 60 yr was used and death at younger age results from experiencing starvation mortality.

Similar to the effect of disturbance, resource seasonality also reduces the maximum reproductive potential of a female by causing pre‐weaned death of calves of young females and reducing female life expectancy (Fig. 3), although it also leads to a younger age at first reproduction. Low to moderate seasonality leads to reduced calf survival and early death of the first one or more calves, while female survival is only slightly affected. At a high level of seasonality, the female is unable to wean any calf and dies at a young age herself (Fig. 3g).

In addition to these direct effects, resource seasonality also aggravates the effect of disturbance and increases the importance of the timing of disturbance. As the seasonal variation in resource density becomes more pronounced, the consequences of summer and winter disturbance begin to diverge. At low to moderate seasonality, disturbance in winter leads to more pre‐weaned deaths of calves compared to summer disturbance (Fig. 3), with no calves being successfully weaned with winter disturbance and a resource amplitude of 0.3. Also, the female suffers from starvation more with winter disturbance and this leads to lower female life expectancy. At high resource seasonality (0.45), the difference between summer disturbance and no disturbance is almost undetectable, while winter disturbance leads to death of the female during the first pregnancy. These different responses arise because in environments with seasonal resource fluctuations, winter disturbance happens when resource density is already low, while summer disturbance occurs in periods when resources are relatively abundant.

In all cases where the calf dies before the age at weaning, it does so early on in lactation. If a calf survives this initial vulnerable period, it is able to withstand successive disturbance events and survives until the age at weaning. This is especially true for calves of older females that have more reserves. In addition, older calves also carry more reserves themselves and have the ability to feed on the resource independently when the mother ceases milk supply.

### Overview of disturbance effects

**Figure 4:**
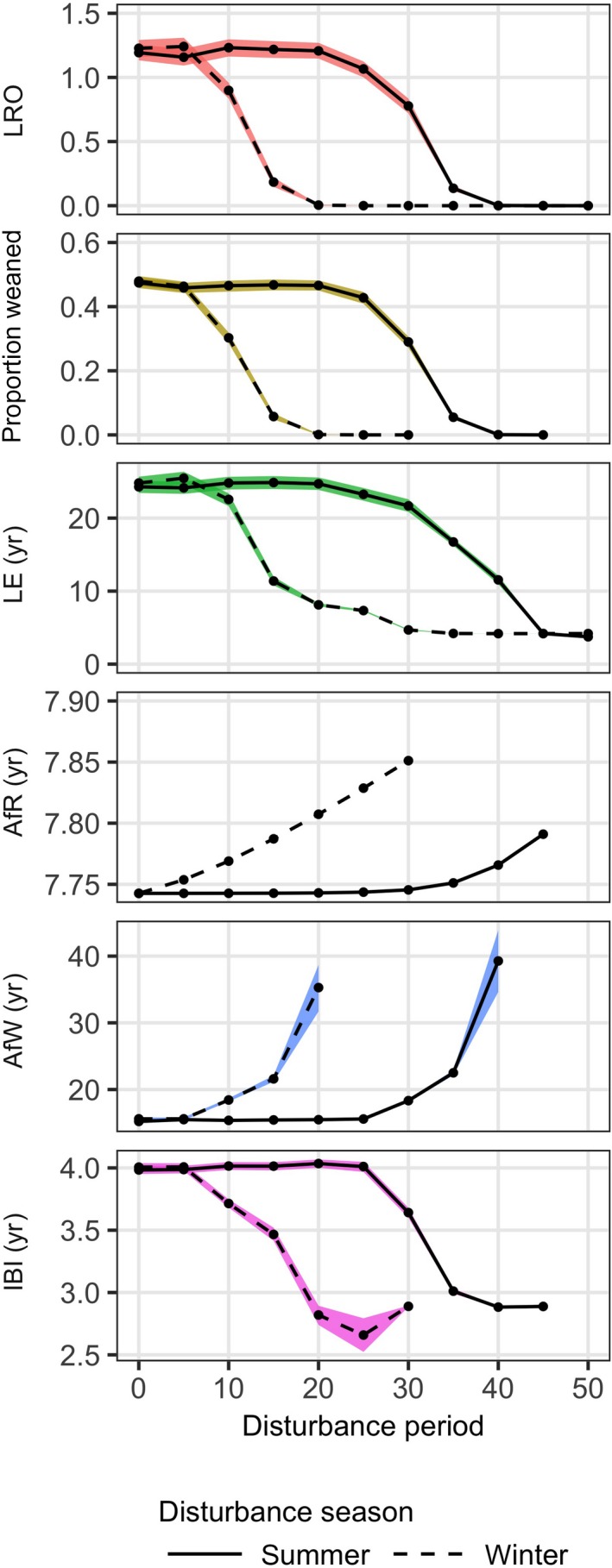
Life history statistics as a function of disturbance period for both summer and winter disturbance. Each data point represents the mean of 1,000 life history simulations, each with a randomly determined life expectancy for both the female and each calf. Colors indicate bootstrapped 95% confidence intervals of the mean. LRO, lifetime reproductive output; Proportioned weaned, proportion of calves that survive until weaning age; LE, life expectancy; AfR, age at first reproduction; AfW, female age at which first calf is weaned; and IBI, inter‐birth interval. The randomly determined life expectancy does not impose variation in age at first reproduction and for some data points at high disturbance values the lack of color bands indicates the coincidence of minimum and maximum values. Mean annual resource density $\hat{R}=2.0$ and resource seasonality *A* = 0.3. All other parameters at default values (Appendix S1: Table S1).

With a resource seasonality of 0.3, the disturbance duration that leads to population decline (*R*_0_ < 1) is approximately 3.3 times higher when disturbance happens in summer, compared to when disturbance happens in winter (Fig. 4, with $\hat{R}=2.0$). With winter disturbance, mean female lifetime reproductive output, mean proportion of weaned calves and mean female life expectancy decline if disturbance duration exceeds 5 d/yr. For summer disturbance, a decline in these life history statistics only occurs beyond 20 d of disturbance per year. The mean and variance of age at first weaning increase with disturbance duration and this happens more rapidly for winter disturbance compared to summer disturbance. No calves are successfully weaned if disturbance exceeds 20 d in winter, or 40 d in summer. The increasing number of pre‐weaned calf deaths lead to a decrease in the mean inter‐birth interval. The age at first reproduction is only marginally affected by disturbance. Variance in age at first reproduction is zero, since the randomly distributed life expectancy does not affect when the female's reserve mass crosses the pregnancy threshold.

The difference between summer and winter disturbance is most apparent at intermediate levels of resource seasonality. A lack of strong seasonal differences in resource density leads to similar responses between summer and winter disturbance, with a predicted population decrease beyond 25 d of disturbance per year (Appendix S2: Fig. S1). Strong resource seasonality has itself a detrimental effect on lifetime reproductive output by diminishing the proportion of successfully weaned calves. In this case, disturbance will further reduce female life expectancy (Appendix S2: Fig. S1).

**Figure 5:**
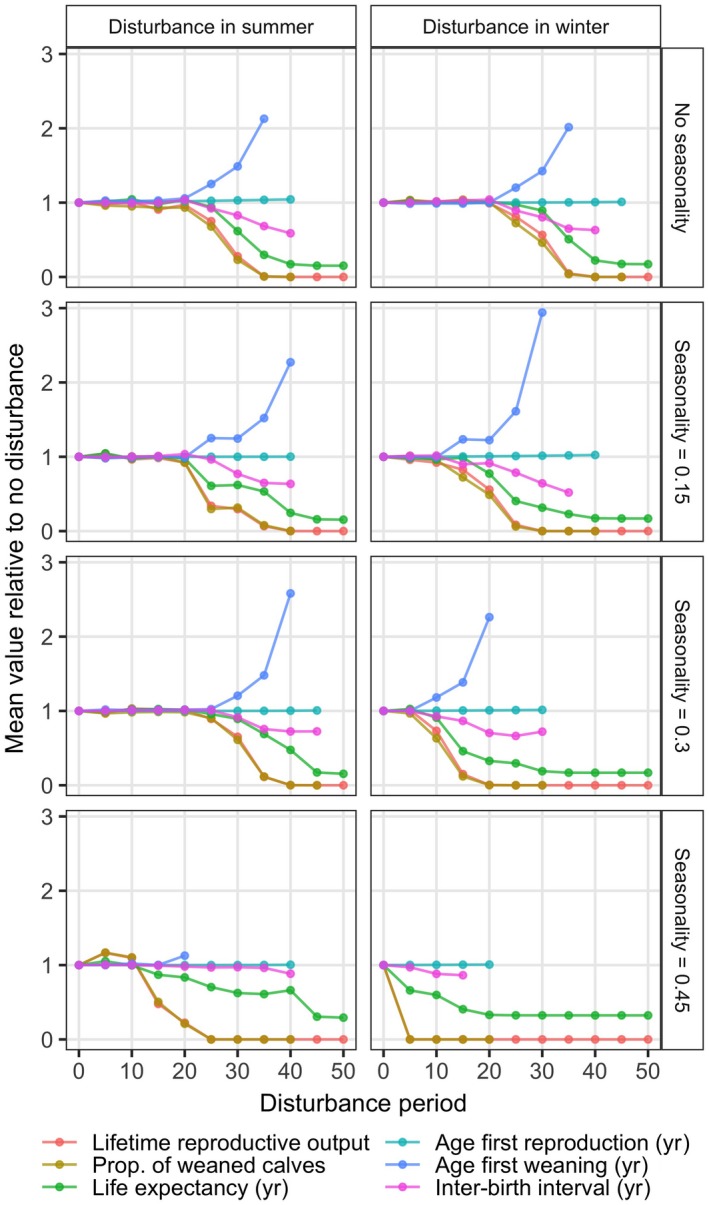
Relative change in the life history statistics as a function of summer (left) and winter (right) disturbance for four levels of resource seasonality (different rows, as indicated in panel labels). These lines are derived by dividing each data point by the mean value at zero days of disturbance, allowing a comparison between the magnitude of change in these life history statistics. Other parameters as in Fig. 4. [Color figure can be viewed at wileyonlinelibrary.com]

The sequence of life history changes with increasing disturbance period is consistent between different levels of resource seasonality and disturbance in winter vs. summer. Using the output from the life history simulations with random life expectancy (Fig. 4; Appendix S2: Fig. S1), we plot the life history statistics relative to the value of each statistic at zero disturbance period in Fig. 5. This shows that changes in the age at first reproduction and the inter‐birth interval are relatively minor. The main change that drives decreasing lifetime reproductive output seems to be the pre‐weaned death of the first few calves, as reflected by the decrease in the proportion of successfully weaned calves and the increase in age at first weaning. Onset of changes in female life expectancy occurs at a slightly higher disturbance duration. However, independent of disturbance season and resource seasonality, the changes in the female's reproductive success with increasing disturbance occur at broadly the same disturbance durations as changes in female survival (Fig. 5).

### Varying resource density

**Figure 6:**
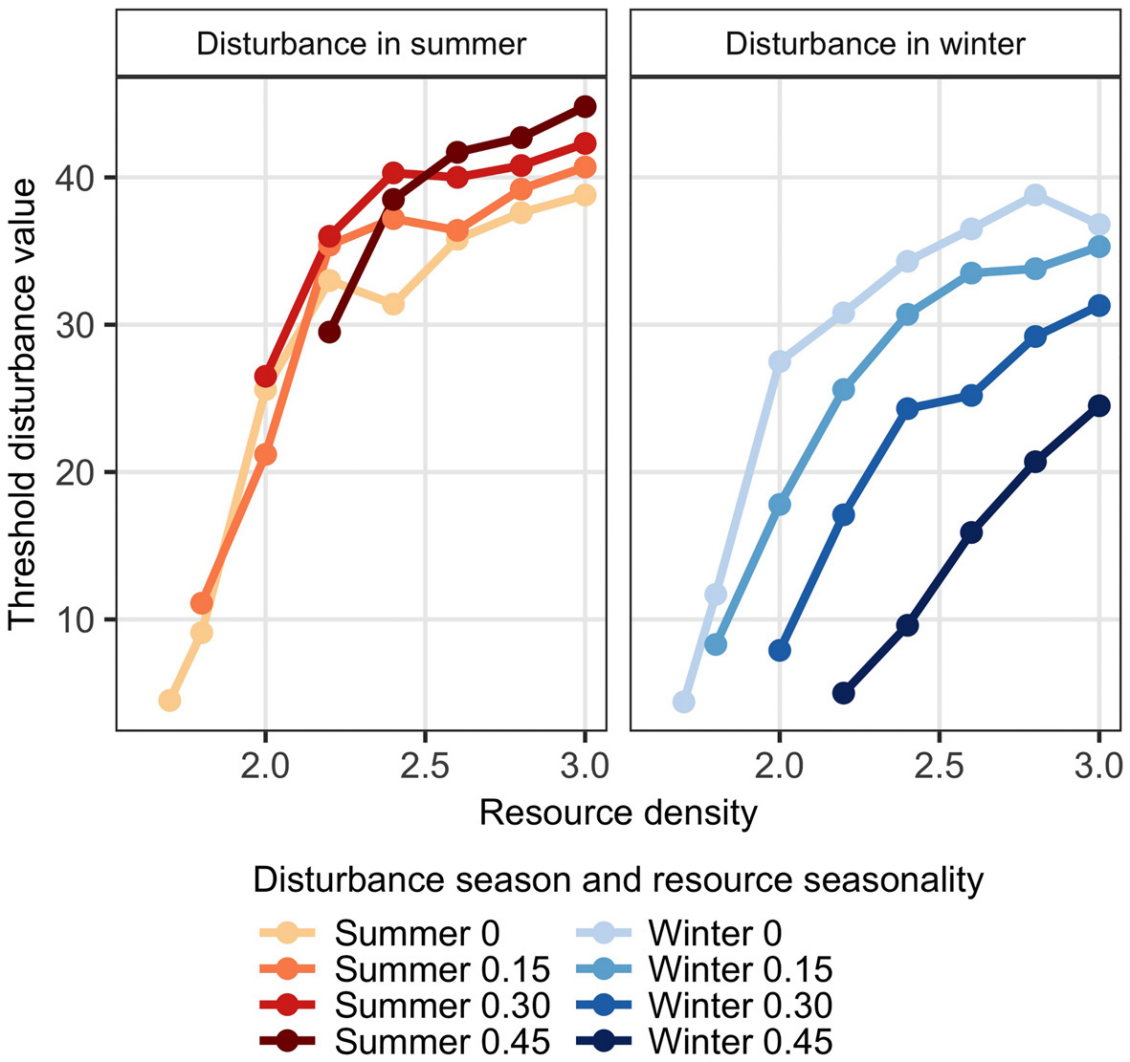
The threshold disturbance value is the disturbance period at which the mean lifetime reproductive output of the female falls below 1 and is plotted as function of mean annual resource density (horizontal axis), for different levels of resource seasonality and either summer or winter disturbance. Higher resource densities allow for longer periods of disturbance. Higher resource seasonality only does so when disturbance happens in summer while in winter resource seasonality increases vulnerability to disturbance.

The differential response to the timing of disturbance relates to the availability of resources to compensate for disturbance. Resource seasonality increases the resource availability in summer, while it decreases resource availability in winter. Consequently, in seasonal environments the female and her calves can only compensate for disturbance when it happens in summer, when resources are relatively abundant. Since the effect of seasonality acts through temporal resource availability, mean resource density will affect this response. We explore the impact of mean resource density by calculating the number of days of lost foraging that is required to negatively impact lifetime reproductive output. This “disturbance threshold value” is quantified by estimating the disturbance period at which the lifetime reproductive output is equal to 1 from a cubic smoothing spline applied to lifetime reproductive output data as a function of disturbance period. Fig. 6 shows how this threshold disturbance value depends on resource density, resource seasonality and the timing of disturbance (summer vs. winter). Overall, mean annual resource density increases the threshold disturbance value in a decelerating manner. Consequently, there exists a limit to which resource density can compensate for disturbance effects. Irrespective of overall resource density, higher levels of resource seasonality require shorter disturbance periods to negatively impact lifetime reproductive output, but only when disturbance happens in winter. When disturbance happens in summer, the effect of resource seasonality varies with resource density. At low mean resource density (<2.5), resource seasonality does not change the threshold disturbance value in any consistent way. However, at high resource density (>2.5), an increase in resource seasonality enables the female to withstand longer periods of disturbance before her lifetime reproductive output falls below 1. Consequently, at high mean annual resource density, resource seasonality aggravates the effect of disturbance in winter, while it attenuates the effect of disturbance in summer.

## Discussion

The model shows that the impact of disturbance crucially depends on resource availability. First, high resource availability compensates for the effect of disturbance, but there is an upper limit to which this is possible. Second, the role of resource availability has important implications with respect to the timing of disturbance, as in many systems resource availability varies seasonally throughout the year, especially in temperate regions. Based on our results, the population can withstand a much longer period of disturbance in periods of abundant food (which we refer to as “summer”) compared to periods of low food (called “winter” in our study). In the most seasonal environment considered here (a relative resource amplitude of 0.45), the disturbance duration that leads to population decline is on average 3.2 times longer for summer disturbance than for winter disturbance (Fig. 6). This ratio between summer and winter disturbance decreases with mean annual resource density, as it equals 5.9 for the lowest resource density and 1.8 for the highest resource density. Field estimates of the level of seasonality in prey availability are required to assess the importance of the timing of disturbance in real‐world systems.

A marked seasonal variation in body fat condition has been observed in Northeast Atlantic pilot whales, with whales being “fat” in winter and “lean” in summer (Lockyer 1993). Body fat content was mainly stored in head and tail muscle, as visceral fat and around visceral organs, while lipid content of the blubber layer varied between 70% and 85% with the lowest level in summer and the highest in winter. This suggests these fat storages mainly serve an energetic purpose. According to Lockyer (1993), the seasonality in body fat condition is probably linked to resource availability, as it is independent of age and reproductive status. This suggests that for Northeast Atlantic pilot whales, resource availability is highest in winter. In general, the phenology of reproductive events such as mating, parturition, and the subsequent onset of lactation might further increase seasonal variation in body condition (Lockyer 2007). During the mating season in early summer (Martin and Rothery 1993), males and females expend additional amounts of energy but may not be able to feed. Our model does not link reproductive events to certain periods within the year, although such an extension could readily be incorporated. Also, we call the season of high resource availability summer, which leads to a peak in body condition during the end of summer and in autumn. The latter choice is of course arbitrary, the important point being that seasonal variation in body condition, either driven by resource availability or phenology of reproductive events, should be taken into consideration when assessing the potential implications of disturbance on wildlife populations.

An increase in thickness of the blubber layer in winter has been observed by Lockyer (1993), indicating that thermoregulatory costs do vary seasonally. Although in the current model we ignore seasonal effects on thermoregulation, there are several ways in which a refined version of the model could account for this. A straightforward possibility is to allow seasonal variation in the target and starvation body condition thresholds. A more challenging alternative is to explicitly model the dynamics of an addition body mass component that represents the blubber layer. However, this latter option would require a good understanding of how these dynamics depend on the multiple functions of the blubber layer (thermoregulation, energy store, buoyancy control) in relation to amounts of reserve and structural mass.

The most extreme form of seasonal variation in body condition are found in long‐distance migratory species, such as baleen whales that do not feed during migration and rely on stored energy to provision migration, lactation and gestation (Alerstam et al. 2003, Stephens et al. 2014). Due to this extreme lifestyle, disturbance affects such species differently compared to a medium‐size cetacean like a pilot whale, which continues to feed throughout the year. For species that rely on stored energy reserves for reproduction and migration, disturbance that leads to cessation of feeding (as studied here) would impact animals only when it happens in the feeding grounds, which is where the energy reserves for the remainder of the migratory cycle are accumulated (Villegas‐Amtmann et al. 2015). Disturbance during migration mainly has consequences when it leads to increased metabolic costs, or separation between mother and calf. Pilot whales feed during the whole year and their fat reserves respond rapidly to environmental conditions. This makes them vulnerable to disturbance that leads to cessation of feeding during periods of low resource availability, and relatively invulnerable to disturbance when resources are high. Based on reserve dynamics in Eq. (1), one might expect that disturbance that increases metabolic costs will have a similar effect as cessation of feeding, as both processes lead to a decrease in available energy for lactation, gestation and growth (Fig. 1). However, one difference between the two forms of disturbance is that compensatory feeding can occur simultaneously with disturbance that increases metabolic costs, but it can only occur after the event if disturbance disrupts feeding.

### The progression of disturbance effects

Independent of resource density or seasonality, increasing the number of days of lost foraging leads to a sequence of changes in the female's life history. As shown in Fig. 5, the changes in age at first reproduction and inter‐birth interval are relatively minor compared to the increase in age at first weaning and the decrease in percentage of successfully weaned calves. This indicates that the initial effect of disturbance that leads to cessation of feeding is to reduce survival of calves produced early in the life of the female. Longer periods of disturbance then lead to a decrease in female life expectancy. While the decrease in reproductive ability, as measured by the proportion of successfully weaned calves, might precede the onset of negative effects of disturbance on survival, the changes in both life history processes occur over a broadly similar range of disturbance durations. As can be seen from Figs. 2 and 3, the negative effects of disturbance on female survival in all cases involves the female crossing the starvation threshold when lactating. Although milk supply ceases at this point, continuing disturbance will inevitably increase starvation mortality and hence decrease life expectancy. Consequently, disturbance results in concurrent effects on female reproduction and survival, because survival is only affected if the female is reproductively active (lactating).

According to our results, young lactating females and their calves are the most sensitive subgroup in the population. When young, the female is still growing and the size of her reserves is limited by her structural capacity. During first lactation, the female in a non‐seasonal environment loses maximally 57 kg of reserves, which equals 35% of the reserve mass at the start of lactation and 9% of total mass (Fig. 2a). In the same environment, a fully grown female loses maximally 45 kg of reserves during lactation (Table 2), equaling 18% of initial reserve mass and 4.6% of total mass. It must be noted, however, that the impact of lactation on reserve mass varies with resource availability. Under high resource availability, reserve mass changes only little with reproductive status. Data from pilot whales catches indicate that pregnant females are heavier than lactating ones, but this relationship was not significant (Lockyer 1993). In North Atlantic right whales, blubber layer was thinner during lactation and then thickened with time after weaning (Miller et al. 2011). Rolland et al. (2016) further discuss trends in body condition of North Atlantic right whales and make a similar classification of female reproductive status as used here, by distinguishing pregnant, lactating, resting, and “available” females (corresponding to our “waiting” category). Similar to our model simulations (Figs. 2 and 3), body condition was higher in available and pregnant females, compared to resting and lactating females (Rolland et al. 2016). This has important implications for monitoring programs that focus on (female) body condition. Poor body condition might actually indicate that females are actively reproducing (lactating or recovering from lactation) and therefore contributing to population growth, rather than indicating that the population suffers from disturbance.

Because responses in the inter‐birth interval and age at first reproduction are relatively small, changes in these life history statistics are likely to remain undetectable in most wildlife populations. Responses in these variables will be more pronounced when reproductive events are restricted to certain periods within the year. This often is the case in species that migrate from feeding to breeding grounds. However, pilot whales are reported to have seasonal reproductive activities (Lockyer 2007). In such cases, disturbance can lead to skipped breeding years if it interrupts mating or if reserve mass is insufficient at the onset of the breeding season. Although reproductive events can occur at any time of year in our model, seasonal resource fluctuations can also induce skipped breeding years and delay the age at first reproduction by one whole year. This mainly occurs for mean annual resource densities lower than the one used in Fig. 4. Incorporating the phenology of reproductive events in the model is expected to increase the occurrence of delayed reproduction and prolong the inter‐birth interval with increasing disturbance duration for higher resource densities.

### Energetics

Lockyer (1993) discusses the energetics of female pilot whales, based on morphometric and biochemical data, which allows a comparison with the outputs from our bioenergetics model (Table 2). Based on the multi‐species equation of Innes et al. (1987), ingestion rate of cetaceans (in kg/d) follows the power function of total body mass 0.123*M* ^0.8^. Applying this formula to the range in total mass of the fully grown female (Table 2) this leads to an ingestion rate of 28–29 kg/d. One of the main prey of pilot whales (the squid *Todarodes sagittatus*) has an energetic content of 4.27 MJ/kg and an assimilation efficiency around 90–95% (Desportes and Mouritsen 1993, Lockyer 1993, 2007). This brings the estimated energy assimilation rate to 108–118 MJ/d, which compares well with our values for pregnant and recovering females (Table 2).

However, the equation from Innes et al. (1987) is based on food intake of captive animals and true ingestion rates are likely to be higher (Bejarano et al. 2017). Resource ingestion rates in our model are largely determined by the field metabolic rate, which is likely an underestimate of the true metabolic rate of pilot whales. We have assumed that field metabolic rate follows a 2.5 multiple from Kleiber's equation for basal metabolic rate, which is an underestimate of the basal metabolic rate of marine mammals (Williams et al. 2001). In addition, Bejarano et al. (2017) compare different estimates of field metabolic rate of bottlenose dolphins (*Tursiops truncatus*) and show that measured daily field metabolic rate is consistently higher than estimated field metabolic rate based on Kleiber's equation with an adjustment factor between 3 and 6. Our presumed underestimate of the field metabolic rate of pilot whales will not affect the model outcome in any qualitative way. The same patterns will occur, but only at a slightly higher mean resource density.

Our model predicts an average 9% and 29% increase in resource assimilation during pregnancy and lactating, respectively. Lockyer (1993) notes that sperm whales increase food intake by 5–10% when pregnant and by 32–62% when lactating, with higher values in growing females. These values compare reasonably well, considering that pilot whales have a longer lactation period than sperm whales (3.35 vs. 2 yr, respectively). Finally, Lockyer (1993) calculates the milk intake during the first year of lactation to be 9,539 MJ, and a corresponding cost of lactation for the female of 11,171 MJ. Although some of the data that lead to these estimates have been used to derive energetic parameters in our model, the similarity of these numbers with our modeled outcomes (9,870 and 11,476 MJ, respectively, Table 2) suggests that the bioenergetics model captures key aspects of pilot whale energetics.

### The choice of disturbance scenarios

The behavioral response to disturbance we have modeled in this paper (complete cessation of feeding for 24 hours) is more extreme than the observed responses of long‐finned pilot whales exposed to military sonar under experimental conditions (Miller 2012, Sivle et al. 2012, Isojunno et al. 2017). However, more extreme responses to actual navy exercises involving sonar have been documented in other medium‐sized cetaceans (e.g., beaked whales; McCarthy et al. 2011, Falcone et al. 2017), and we chose to model an extreme response in order to provide a clear picture of the potential effects of lost foraging opportunities on pilot whale life histories. For similar reasons, we modeled longer disturbance durations as continuous periods during which no foraging was possible. As a result, disturbed animals could not compensate for lost foraging opportunities until all disturbance had ended. In practice, individuals within a population are likely to be exposed to different, time‐varying patterns of disturbance that will probably have a less profound effect on survival and reproduction that we observed in our simulations. The framework described here can be readily adapted to investigate the potential effects of these real‐world disturbances, provided their nature can be accurately described.

## Conclusions

We used a bio‐energetics approach to model the life history of a pilot whale female and her calves. With this model, we study how increasing levels of disturbance that cause cessation of foraging affect female life history and calf survival and how the consequences of disturbance depend on resource availability and its variation through time. Although the model was specifically parameterized and tailored for long‐finned pilot whales, its structure is general enough to represent other income breeding (marine) mammals if appropriate data on life history and energetics are available. In fact, a similar bio‐energetics model with the same general structure (Fig. 1) was used to describe the dynamics of a population of ungulates living in a grassland environment with seasonally varying productivity (De Roos et al. 2009). The same model could therefore be used to provide insights into the general consequences of disturbance. If more detailed information is available, it can be used to provide management advice for specific species or populations.

## Data Availability

Associated code is available on Zenodo: https://doi.org/10.5281/zenodo.2605195

## Note

1 https://staff.fnwi.uva.nl/a.m.deroos/EBT/Software/index.html
