# Supplementary material for: Bio‐energetic modeling of medium‐sized cetaceans shows high sensitivity to disturbance in seasons of low resource supply
Source: Ecol Appl. 2019 May 16;29(5):e01903. doi: 10.1002/eap.1903 (PMC6618318; doi:10.1002/eap.1903)
Supplement: Supplementary file 1 [file EAP-29-na-s001.pdf]

Supporting Information. Hin, Vincent, John Harwood, and André M. de Roos. 2019. Bio-energetic modeling of medium-sized cetaceans shows high sensitivity to disturbance in seasons of low resource supply. *Ecological Applications*.

Appendix S1: Model Parameters

Most model parameters are derived from the international research program on Northeast Atlantic long-finned pilot whales off the Faroe Islands, which was conducted between July 1986 and July 1988 (Bloch et al. 1993*b*). The year-round drive-fisheries on the Faroe Islands resulted in detailed information on age and growth parameters (Bloch et al. 1993*a*), reproductive parameters (Martin and Rothery 1993) and bioenergetic parameters (Lockyer 1993) of a large number of pilot whales (n > 3000). Some parameter values could not be derived directly from sources (*e.g.* lactation scalar ϕ_L) and these were based on reasonable biological assumptions and the values of other parameters. All model parameters with references to the sources used in their derivation are listed in Table S1.

Estimates of gestation and lactation periods are given by Martin and Rothery (1993) and amount to 12 months (365 days) and 40.2 months (1223 days), respectively. Lockyer (1993) suggests that during the first year of life, calves are solely dependent on milk, which gives $T_N = 365$. The duration of the waiting period was calculated by assuming that ovulation occurs once per year and has a chance of pregnancy of 82%. This latter chance is derived from the ratio between the inter-ovulation interval and the inter-birth interval (4.17 : 5.1, as reported by Martin and Rothery 1993). This leads to a waiting period of $\frac{365}{0.82} = 445$ days. Estimates for the parameters describing the increase in resource feeding efficiency with age (T_R and γ) are not available. The age at which this function equals 0.5 is controlled by parameter T_R, while the non-linearity is determined by γ. By default, we adopt $T_R = 500$ and $\gamma = 2$. With these values, the resource feeding efficiency increases rapidly at first, equals ~ 86% per cent at weaning age and asymptotically approaches 100% (Figure S1c).

Bloch et al. (1993a their Table 7) report several estimates for length at birth and total mass at birth and we adopt their best estimates of $l_b = 177$ cm and $W_b = 75$ kg. Mean length-at-age data in Bloch et al. (1993a their Table 10) is used to fit parameters l_∞ and k. In the fitting procedure, its assumed that the reported ages indicate the beginning of the age classes, which implies that the true mean ages are 0.5 years later. This age-transformation resulted in a satisfactory fit of a non-linear regression with parameters $l_\infty = 450$ and $k = 0.00045$, while fixing $l_b = 177$ (Figure S1a).

Energy densities for pilot whales at different ages as reported by Lockyer (1993) were used to estimate the length-structural mass relationship parameters ω_1 and ω_2, and the target and

starvation body condition parameters ρ and ρ_s. In this derivation, we use lipid proportion as an indication of the percentage of reserve mass of an individual. The fact that a part of the lipid content of an individual has a structural origin and cannot be mobilized without compromising survival is accounted for by setting the starvation threshold $\rho_s > 0$. Lipid proportion for calves is around 17% (Lockyer 1993), which makes structural mass at birth $S_b = 75 \cdot (1 - 0.17) = 62$ kg. Ultimate body mass is reported by Bloch et al. (1993a) as $W_\infty = 1320$ kg for individuals with $l_\infty = 512$ cm. According to Lockyer (1993), lipid content of these individuals is around 25%, so by a similar calculation $S_\infty = 990$ kg. This leads to a length-structural mass scaling exponent of $\omega_1 = \ln\left(\frac{S_\infty}{S_b}\right)/\ln\left(\frac{l_\infty}{l_b}\right) = 2.6$. Consequently, the length-structural mass scaling constant equals $\omega_2 = 8.5 \cdot 10^{-5}$. A large proportion of the lipid content of calves (17%) probably belongs to structural mass and cannot be used as energy reserve without endangering life. Based on this we adopt $\rho_s = 0.15$. This corresponds well to the observation that most pilot whales of the Faroese population have a body condition in the range 0.14 – 0.23 (Lockyer 1993). Since modeled reserve mass for females equilibrates below the target reserve threshold, we adopt $\rho = 0.30$. The steepness of the change in feeding effort with body condition is controlled by parameter η. Since there are no empirical estimates of this function, we adopt $\eta = 15$ by default.

Compared to the maintenance costs for structural mass, the maintenance costs of reserves are lower per unit body mass. This relative discounting is quantified with parameter θ_F. Dynamic Energy Budget (DEB) theory argues that reserve mass does not require maintenance (Kooijman 2010), which implies that $\theta_F = 0$. While the amount of basal metabolic costs of reserves might be limited, reserves can increase field metabolic costs by increasing energetic costs of locomotion and activity. This holds especially for marine mammals, in which large fat reserves can increase drag. We therefore adopt the conservative estimate of $\theta_F = 0.2$. Although maintenance costs of fetus mass might also be lower per unit mass than maintenance costs of structural mass, fetal maintenance also includes costs for the placenta as well as increased metabolic costs for the mother due to increased drag and activity costs. Consequently, no discounting for fetal metabolism was applied.

Field metabolic maintenance rate follows Kleiber's relationship (Kleiber 1975). The scalar of this relationship (σ_M) was set to 0.75. This approximately represents a 2.5 multiple of basal metabolic rate, as was also adopted by Lockyer (1993). Costs of growth in structural mass (represented by σ_G) includes both growth overheads and the energy assembled in the newly synthesized biomass. We derive an estimate for σ_G by combining Brody's (1968) equation for the heat of gestation ($Q_G = 4400W(0)^{1.2}$ in kCal) with estimates of the energy densities of pilot whales as reported by Lockyer (1993). Heat of gestation for a 75 kg neonate amounts to 3274 MJ. This includes the fetal maintenance metabolic rate during the gestation period, which in our model amounts to $\int_0^{T_P} \sigma_M \left(\omega_1 \left(l_b \frac{\tau_P}{T_G}\right)^{\omega_2}\right)^{0.75} d\tau_c = 1987$ MJ. Energy content of a 75 kg neonate is estimated to be 7.95 · 75 · 1.25 = 745 MJ. This estimate accounts for the placenta, which adds

25% to the neonate mass and uses an energy density of fetus and placenta at birth of 7.95 MJ/kg (Lockyer 1993). This implies a growth efficiency of 745 / (3274 – 1987 + 745) = 0.366. Pilot whale calves have a considerably higher energy density of 10.1 MJ/kg (Lockyer 1993), which results in $\sigma_G = 27.4$ MJ/kg. Since the heat of gestation is likely to be an underestimate of the true costs of gestation we settle for a value of $\sigma_G = 30$ MJ/kg.

The conversion efficiency parameter σ_L controls both the assimilation efficiency of milk by calves and the efficiency with which the mother produces milk from reserves. Lockyer (1993) assumes that efficiency of milk assimilation is 95% and that mammary gland efficiency of milk production is 90%. Combining these estimates yields a value of $\sigma_L = 0.86$.

The lactation scalar ϕ_L was parameterized such that the amount of energy expended in the first year of life (maintenance and growth costs) can be completely covered by milk suckling, which seems reasonable for pilot whales (Lockyer 1993). Energy expended on maintenance during the first year follows from solving the integral: $\int_0^{365} \sigma_M \left[\left(1 + \theta_F \frac{\rho}{1-\rho}\right) \omega_1 l(a)^{\omega_2}\right]^{0.75} da = 7808$ MJ, in which it is assumed that $F/W = \rho$ and that $l(a)$ follows the Von Bertalanffy length-age relationship. Using the relationship between age and structural mass, the structural growth during the first year equals 41 kg and corresponding growth costs amount to 1230 MJ. If milk assimilation covers all these expenses then, assuming $F/W = \rho$ for both mother and calf, the milk ingestion rate function I_L evaluates to $9038/365 = \phi_L \cdot S^{2/3} \cdot 0.5$. Using an average structural mass of the calf in the first year of $S = \left(\int_0^{365} \omega_1 l(a)^{\omega_2} da\right)/365 = 80.5$ kg, gives $\phi_L = 2.7$.

Parameters describing the efficiency of anabolic and catabolic reserve dynamics (ε_+ and ε_-) were set to 55 MJ/kg and 35 MJ/kg, respectively. The efficiency of catabolism closely relates to the energy density of fat, which was reported as 40 MJ/kg (Lockyer 1993). Assuming a roughly 90% efficiency of catabolic conversion leads to the value of 35 MJ/kg. Anabolic conversion is considered less efficient and we hence set ε_+ to 55 MJ/kg.

The parameters ξ_c and ξ_m describe the non-linearity of milk assimilation with calf age and female body condition, respectively. Empirical estimates for these parameters do not exist for any marine mammal species. We therefore choose these parameters such that their functions reflect the biological response in a qualitative manner. The parameter ξ_m described how milk supply of the female depends on her body condition and is set to -2, to simulate a decline in the rate of milk provisioning that decreases with decreasing body condition of the female. The parameter ξ_c controls the steepness of the decrease in milk assimilation rate with increasing age of the calf. A value of $\xi_c = 0.9$ ensures that milk suckling decreases with an increasing rate as the calf ages.

Mortality rate parameters are derived from Bloch et al. (1993a), who fit a survivorship curve to an age-frequency plot of Pilot Whales from the Faroe Islands (n = 1,482 Bloch et al. 1993a their Figure 3) and present age-specific survival estimates based on the method of Barlow and Boveng (1991). We use these age-specific survival estimates (P_a) to calculate the age-specific mortality rate as $z_a = -\ln(P_a)$. Subsequently, we fit the age-dependent mortality rate function to the age-specific mortality rates z_a, to obtain the estimates of the parameters α_1, α_2, β_1 and β_2 as shown in Table S1. We set μ_s to 0.2, which implies a starvation mortality rate of 0.1 day^{-1} for an individual with a body condition of 0.1 and (ignoring age-dependent mortality) a 50% survival over a period of one week of starvation if body condition remains at 0.1.

Literature Cited

Barlow, J., and P. Boveng. 1991. Modeling Age-Specific Mortality for Marine Mammal Populations. Marine Mammal Science 7:50–65.

Bloch, D., C. Lockyer, and M. Zachariassen. 1993a. Age and Growth Parameters of the Long-Finned Pilot Whale off the Faroe Islands. Report of the International Whaling Commission, Special Issue 14:163–207.

Bloch, D., G. Desportes, R. Mouritsen, S. Skaaning, and E. Stefansson. 1993b. An Introduction to Studies of the Ecology and Status of the Long-finned Pilot Whale (*Globicephala melas*) off the Faroe Island, 1986 – 1988. Report of the International Whaling Commission, Special Issue 14:1–32.

Brody, S. 1968. Bioenergetics and Growth. Hafner Publishing Co., New York.

Kleiber, M. 1975. The fire of life: an introduction to animal energetics. R.E. Krieger Pub. Co., Huntington, N.Y.

Kooijman, S. A. L. M. 2010. Dynamic Energy Budget theory for metabolic organisation. Third edition. Cambridge University Press, Cambridge, UK.

Lockyer, C. 1993. Seasonal Changes in Body Fat Condition of Northeast Atlantic Pilot Whales, and their Biological Significance. Report of the International Whaling Commission, Special Issue 14:325–350.

Martin, A. R., and P. Rothery. 1993. Reproductive Parameters of Female Long-Finned Pilot Whales (*Globicephala melas*) Around the Faroe Islands. Report of the International Whaling Commission, Special Issue 14:263–304.

Table S1: Model Parameters

Symbol	Unit	Value	Description	Source
$\boldsymbol{T_P}$	day	365	Gestation period	Martin & Rothery 1993
$\boldsymbol{T_L}$	day	1223	Lactation period (age at weaning)	Martin & Rothery 1993
$\boldsymbol{T_N}$	day	365	Age at which milk consumption starts to decrease	–
$\boldsymbol{T_R}$	day	500	Age at which resource foraging is 50%	–
$\boldsymbol{T_D}$	day	445	Waiting period before onset of pregnancy	Martin & Rothery 1993
$\boldsymbol{l_b}$	cm	177	Length at birth	Martin & Rothery 1993
$\boldsymbol{l_\infty}$	cm	450	Ultimate length in Von Bertalanffy Growth Curve	Bloch et al. 1993; this study
$\boldsymbol{k}$	day^{-1}	0.00045	Von Bertalanffy growth rate	Bloch et al. 1993; this study
$\boldsymbol{\omega_1}$	kg/cm^{ω_2}	$8.5 \cdot 10^{-5}$	Structural mass-length scaling constant	Bloch et al. 1993; this study
$\boldsymbol{\omega_2}$	–	2.6	Structural mass-length scaling exponent	Bloch et al. 1993; this study
$\boldsymbol{\theta_F}$	–	0.2	Relative metabolic cost of reserves	–
$\boldsymbol{\rho}$	–	0.30	Target body condition threshold	Lockyer 1993
$\boldsymbol{\rho_s}$	–	0.15	Starvation body condition threshold	Lockyer 1993
$\boldsymbol{\phi_R}$	$m^3 \cdot kg^{-2/3} \cdot day^{-1}$	1.0	Resource encounter rate scalar	–
$\boldsymbol{\phi_L}$	$MJ \cdot kg^{-2/3} \cdot day^{-1}$	2.7	Lactation scalar	Lockyer 1993; this study
$\boldsymbol{\eta}$	–	15	Steepness of assimilation response around target body condition	–
$\boldsymbol{\gamma}$	–	2	Shape parameter of resource assimilation-age response	–

$\boldsymbol{\xi_m}$	–	–2.0	Non-linearity in female body condition-milk provisioning relation	–
$\boldsymbol{\xi_c}$	–	0.9	Non-linearity in milk assimilation-calve age relation	–
$\boldsymbol{\sigma_M}$	$MJ \cdot kg^{-3/4} \cdot day^{-1}$	0.75	Field metabolic rate scalar	Lockyer 1993
$\boldsymbol{\sigma_G}$	$MJ \cdot kg^{-1}$	30	Energetic costs per unit structural mass growth	this study
$\boldsymbol{\sigma_L}$	–	0.86	Lactation conversion efficiency	Lockyer 1993
$\boldsymbol{\alpha_1}$	day^{-1}	$4.01 \cdot 10^{-4}$	Mortality parameter	Bloch et al. 1993; this study
$\boldsymbol{\beta_1}$	day^{-1}	$5.82 \cdot 10^{-4}$	Mortality parameter	Bloch et al. 1993; this study
$\boldsymbol{\alpha_2}$	day^{-1}	$6.04 \cdot 10^{-6}$	Mortality parameter	Bloch et al. 1993; this study
$\boldsymbol{\beta_2}$	day^{-1}	$3.01 \cdot 10^{-4}$	Mortality parameter	Bloch et al. 1993; this study
$\boldsymbol{\mu_s}$	day^{-1}	0.2	Starvation mortality scalar	–
$\boldsymbol{\varepsilon_+}$	MJ/kg	55	Anabolic reserves conversion efficiency	–
$\boldsymbol{\varepsilon_-}$	MJ/kg	35	Catabolic reserves conversion efficiency	Lockyer 1993
$\boldsymbol{\hat{R}}$	MJ/m^3	1.6 – 3.0	Mean annual resource density	–
$\boldsymbol{A}$	–	0, 0.15, 0.3, 0.45	Relative amplitude of seasonal resource fluctuation	–

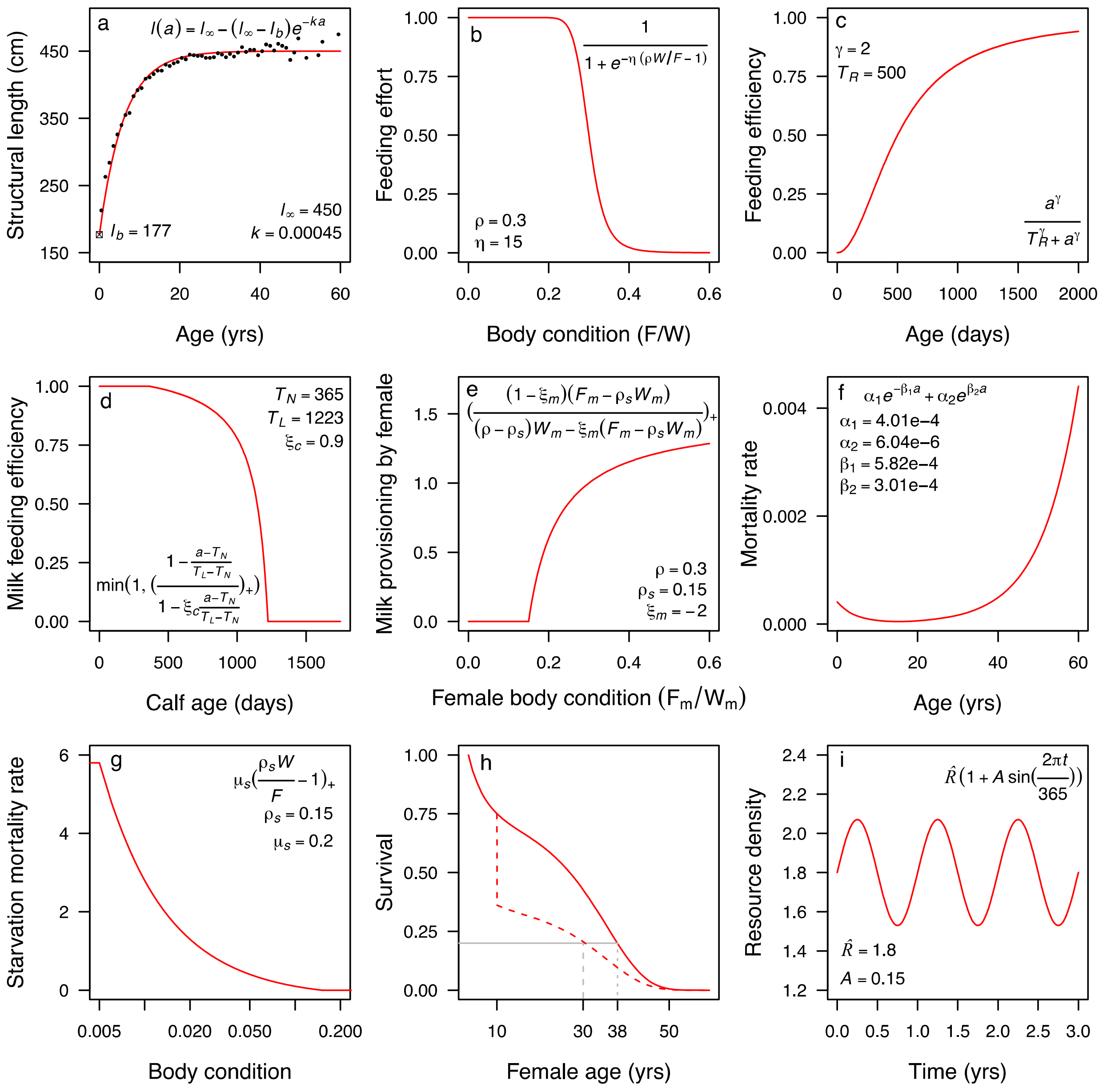

Figure S1: An overview of nine different model components and their associated functions and parameters. Some components are applied within the same model function. For example, panels b, d and e all appear in the function $I_L(\ldots)$, that describes milk assimilation rate. The variables age a and time t are always in units of days, but for convenience sometimes plotted in years. Panel a shows data from Bloch et al. (1993a) on which the length-age relationship is parameterized with small solid points and the initial length at birth estimate as a crossed open square. Panel h shows how mortality decreases survival for the female that is initiated at weaning age with a solid curve. Additionally, the dashed curve illustrates a particular scenario in which survival is affected by 15 days of starvation mortality at an age of 10 years. The gray lines in panel h indicate how starvation mortality reduces the life expectancy from 38 to 30 years. Subscript '+' indicates positive values only, otherwise zero.
