# Supplementary material for: Bio‐energetic modeling of medium‐sized cetaceans shows high sensitivity to disturbance in seasons of low resource supply
Source: Ecol Appl. 2019 May 16;29(5):e01903. doi: 10.1002/eap.1903 (PMC6618318; doi:10.1002/eap.1903)
Supplement: Supplementary file 2 [file EAP-29-na-s002.pdf]

Supporting Information. Hin, Vincent, John Harwood, and André M. de Roos. 2019. Bio-energetic modeling of medium-sized cetaceans shows high sensitivity to disturbance in seasons of low resource supply. *Ecological Applications.*

Appendix S2

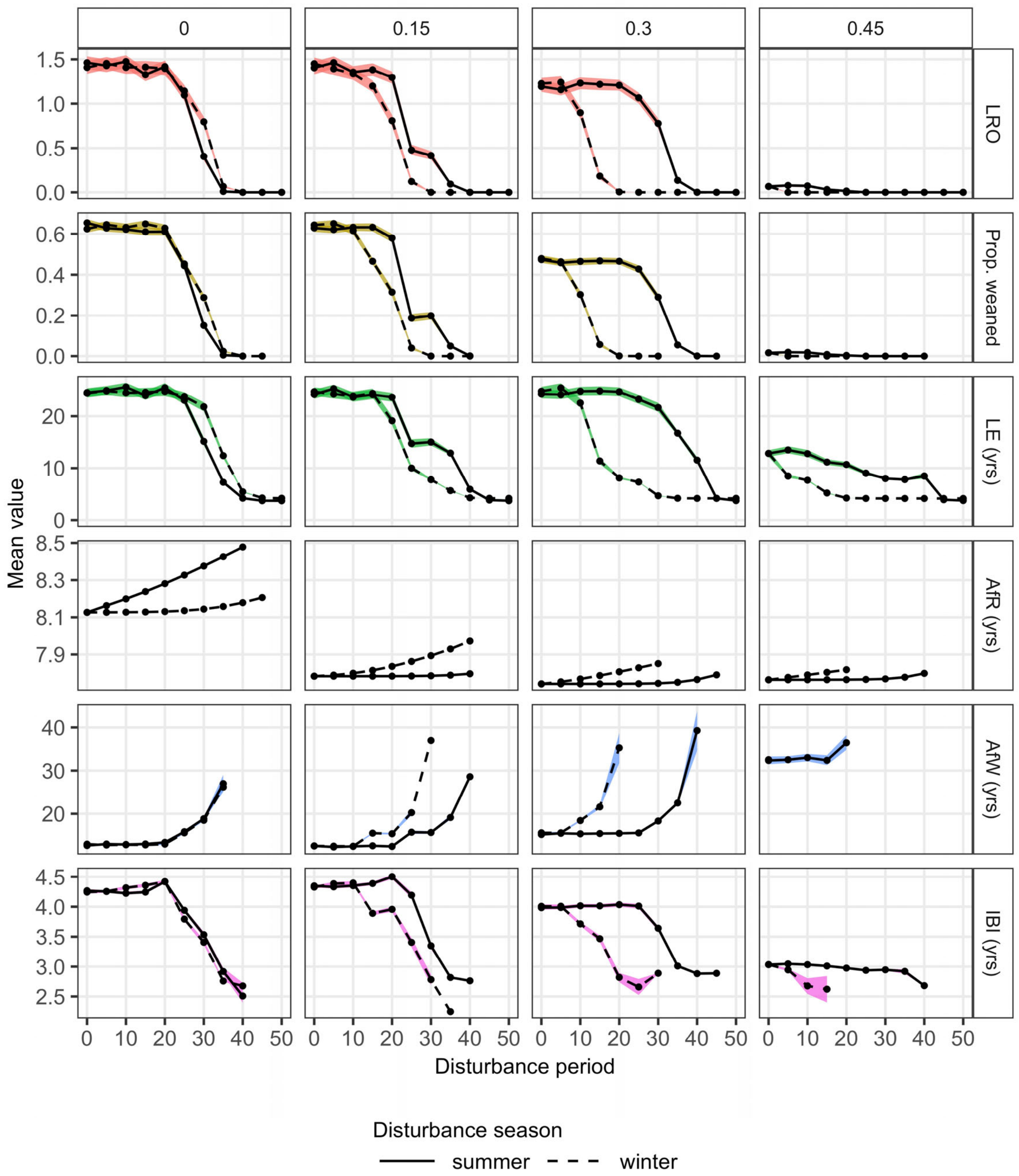

Figure S1: Life history statistics as a function of disturbance period for both summer and winter disturbance and four different levels of resource seasonality (different columns). Each data point represents the mean of 1,000 life history simulations, each with a randomly determined life expectancy for both the female and each calf. Colors indicate bootstrapped 95% confidence intervals of the mean. LRO: lifetime reproductive output, Prop. weaned: proportion of calves that survive until weaning age, LE: life expectancy, AfR: age at first reproduction, AfW: female age at which first calf is weaned and IBI: inter-birth interval. The randomly determined life expectancy does not impose variation in age at first reproduction and for some data points at high disturbance values the lack of color bands indicates the coincidence of minimum and maximum values. Mean annual resource density $\hat{R} = 2.0$ and all other parameters at default values (Appendix S1: Table S1).
